# Structural Evolution of Stapes Controls the Electrochemical CO_2_ Reduction on Bimetallic Cu‐doped Gold Nanoclusters

**DOI:** 10.1002/smll.202408531

**Published:** 2024-12-02

**Authors:** Enric Ibáñez‐Alé, Jiajun Hu, Josep Albero, Laura Simonelli, Carlo Marini, Núria López, Noelia Barrabés, Hermenegildo García, Sara Goberna‐Ferrón

**Affiliations:** ^1^ Institute of Chemical Research of Catalonia, (ICIQ‐CERCA) The Barcelona Institute of Science and Technology (BIST) Av. Països Catalans 16 Tarragona 43007 Spain; ^2^ Universitat Rovira i Virgili Avinguda Catalunya, 35 Tarragona 43002 Spain; ^3^ Institution Instituto Universitario de Tecnología Química (CSIC‐UPV) Universitat Politècnica de València Avda. De los Naranjos s/n Valencia 46022 Spain; ^4^ ALBA Synchrotron Light Facility Carrer de la Llum 2‐26 Cerdanyola del Valles 08290 Barcelona Spain; ^5^ Institute of Materials Chemistry Technische Universität Wien Getreidemarkt 9/BC/01 Vienna 1060 Austria

**Keywords:** CO_2_ electroreduction, doping, gold nanoclusters, ligand, mechanism

## Abstract

Ligand protected gold nanoclusters have been proposed for electrochemical CO_2_ reduction (eCO_2_R) as an alternative to polycrystalline catalysts, showing higher selectivity control due to the tailored composition and precise microenvironment. Here, two gold cluster families are studied with different staple motifs (Au_25_(SR)_18_ and Au_144_(SR)_60_, where SR = thiolate) doped with Ag or Cu to understand the interplay between the composition and the performance of these catalysts. Detailed cluster characterization and Density Functional Theory simulations demonstrate that the dynamic aspects involving ligand removal are crucial to unraveling the role of the dopant, the cluster curvature, and the staple structure. The best activity performance toward CO is obtained for Cu‐doped Au_144_(SR)_60_ at *U* = –0.8 V_RHE_ as ligands are only partially depleted and the staple can bend to stabilize *CO intermediate, while Cu‐containing Au_25_(SR)_18_ can produce formate but shows worse CO selectivity. This study points toward the importance of ligand stability during eCO_2_R on bimetallic gold nanoclusters, paving the way for improving the design and operation of this family of catalysts.

## Graphical abstract

While larger Au_144_ clusters can give high conversions for eCO_2_R to CO and HCOOH, their catalytic performance will decay at strong cathodic potentials due to high ligand depletion. On the other hand, Au_25_ clusters can retain part of the ligands even at these cathodic potentials, which enables a more stable catalytic performance.

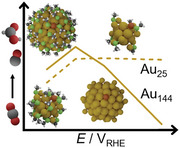

## 1 Introduction

Turning CO_2_ emissions into fuels or value‐added chemicals, such as carbon monoxide (CO), formic acid, methanol, and methane, represents an important opportunity for CO_2_ utilization and valorization.^[^ ^1^ ^]^ Of the existing approaches, electrochemical CO_2_ reduction (eCO_2_R) is a promising strategy since it takes place under mild conditions, and the reaction parameters can be easily controlled.^[^ ^2^, ^3^ ^]^ However, achieving high selectivity and efficiency toward the desired products remains challenging. For this purpose, metallic catalysts with molecule‐like properties and well‐defined monodisperse particle sizes have shown improved catalytic performance compared to their bulk counterpart.^[^ ^4^ ^]^ Among them, atomically precise ligand‐protected metal nanoclusters (NCs) are defined as sub‐3 nm particles surrounded by ligands of different sizes and compositions. They have received enormous attention because of their unique chemical and physical properties with applications in catalysis, photovoltaics, photosensitizers, chemical sensors, nanodevices, etc.^[^ ^5^, ^6^, ^7^, ^8^, ^9^, ^10^ ^]^ Due to the atomically precise composition and defined atom packing and microenvironment, these nanoclusters facilitate the straightforward correlation between structure and catalytic properties. Metal NCs, primarily Au‐, Ag‐, and Cu‐based NCs, stabilized by various ligands including thiolate, phosphine, carbene, alkynyl, and amidinate, thus with different microenvironments, have been investigated for eCO_2_R studies.^[^ ^11^, ^12^, ^13^ ^]^ In contrast to gold and silver, copper NCs remain challenging due to the lower susceptibility to reduction of Cu^I^ precursors and the higher susceptibility to oxidation of as‐formed products.^[^ ^14^, ^15^ ^]^ Therefore, reducing Cu^I^ precursors with a hydride source often results in the synthesis of stable Cu^I^ hydride clusters.^[^ ^16^, ^17^, ^18^, ^19^ ^]^ Nevertheless, the presence of interstitial H^−^ ions arranges the Cu atoms in the cluster very differently from those packed in actual Cu NPs or even metallic Cu. Despite these challenges, Cu nanomaterials are of intense interest in eCO_2_R as a unique metal catalyst that can yield a variety of products by multi‐electron reduction.^[^ ^20^ ^]^ In this regard, alloying is an efficient way to improve the stability and catalysis performance of metal NCs.^[^ ^21^, ^22^, ^23^, ^24^, ^25^, ^26^, ^27^ ^]^ For instance, the first report of C_2+_ production from eCO_2_R on metal NCs was published very recently using fcc‐structured bimetallic (AgCu)_50_ NCs protected by thiolate ligands as catalysts.^[^ ^28^ ^]^

Among these types of materials, thiolate‐protected Au NCs are one of the most widely studied metal NCs that have been utilized to catalyze eCO_2_R.^[^ ^29^ ^]^ Specifically, Au_25_(SR)_18_ holds an exceptional place in this field because of easy preparation, high stability, and thus easy functionalization and application.^[^ ^30^ ^]^ Since the discovery of the electrocatalytic activity of Au_25_(SR)_18_ for CO_2_ to CO reduction in 2012,^[^ ^31^ ^]^ a plethora of studies have dealt with metal‐doping,^[^ ^21^, ^23^, ^24^, ^25^, ^32^, ^33^ ^]^ morphology,^[^ ^34^ ^]^ protecting ligands,^[^ ^35^ ^]^ or mechanistic aspects.^[^ ^36^, ^37^, ^38^, ^39^, ^40^ ^]^ As for metal heteroatoms, Ag, Cd, and Pd incorporated in Au_25_(SR)_18_ NCs tend to improve eCO_2_R activity and selectivity towards CO. However, Pt has shown enhanced hydrogen evolution reaction (HER) selectivity. Surprisingly, although it has been demonstrated that Cu can be doped into Au_25_(SR)_18_ NCs^[^ ^41^, ^42^, ^43^, ^44^ ^]^ affecting their electrocatalytic activity,^[^ ^33^, ^45^ ^]^ to the best of our knowledge, the exact role of Cu‐substitution on the CO_2_ reduction performance has not yet been reported.

Regarding mechanistic aspects, the stability of ligands should be taken into account as they are commonly considered to be partially removed at reductive potentials which can expose the active sites on inner NCs kernel. For thiolate‐protected Au NCs, there are mainly two viewpoints on the reaction active sites: i) dethiolation (removal of −SR) to expose kernel Au active sites, and ii) dealkylation (removal of −R) to expose staple Au−S active sites.^[^ ^38^ ^]^ In the case of Au_25_(SR)_18_, DFT investigations demonstrated that the dethiolated Au site generated in each −SR−Au−SR−Au−SR− terminal staple motif (Au···SR−) exhibited a relatively high charge density and upshift of *d*‐states, which facilitated the eCO_2_R process by stabilizing the *COOH intermediate.^[^ ^39^ ^]^ On the other hand, it has been proposed that *CO adsorption on the S active site can cause S−Au bond rupture (and further regeneration) from the Au−S−Au staple motif in Pd‐doped and undoped Au_25_(SR)_18_ NCs and the S−Au and Cd−S bond rupture in the Cd−S−Au staple motif in the Cd‐doped Au_23_(SR)_16_ NC, which could better stabilize the *CO species in contrast to the intact Au−S and Cd−S bonds.^[^ ^21^, ^22^ ^]^ Moreover, an enhanced CO selectivity due to ligand stabilization induced by Cd‐doping has been reported, suggesting improved eCO_2_R if ligands on staples, thus blocking kernel Au sites that enhance competing HER.^[^ ^25^ ^]^ However, the metal NCs features controlling the reaction mechanisms remain important since factors such as geometric structures, staple structure evolution, and ligand stability effects are not yet entirely understood.^[^ ^39^, ^40^ ^]^ Furthermore, the evolution of the staple can undergo different pathways, including fragmentation or migration to the support material, as documented in previous studies.^[^ ^46^, ^47^, ^48^, ^49^ ^]^ Despite the well‐defined structure of larger NCs as Au_144_(SR)_60_^[^ ^50^, ^51^ ^]^ enables the study of higher geometric structures but shorter staple motifs, the evolution of its ligands upon reaction conditions, and how they affect the eCO_2_R performance has not been explored yet. Therefore, despite the large research efforts on bimetallic NCs as eCO_2_R catalysts, stability issues have been avoided.

In this study, we explore the impact of ligand evolution on electrochemical CO_2_ reduction through a combined experimental and theoretical approach. Using well‐known thiolate‐protected clusters like Au_25_(SR)_18_ and Au_144_(SR)_60_ with distinct staple motifs, we introduce Cu and Ag as doping atoms to investigate their influence. The nanocluster catalysts were characterized using various techniques, including X‐ray absorption fine structure (XAFS), before and after the reaction. The integrated analysis, along with catalytic activity and Density Functional T−heory (DFT) simulations, enable us to find for each cluster an optimal potential before dethiolation starts to hinder eCO_2_R, with both Au_25_(SR)_18_ and Au_144_(SR)_60_ showing the best CO selectivity at −0.7 V_RHE_. Thus, the observed decrease of CO production at strong reductive potentials suggests that ligands are sacrificed, consuming a significant amount of the current that compromises the catalytic performance of Au NCs.

## 2 Results and Discussion

**Scheme 1:**
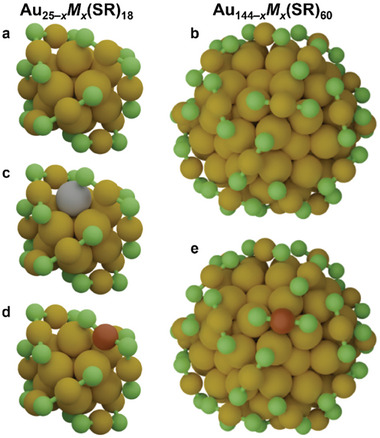
Structure representation of synthesized a) Au_25_(SR)_18_ clusters and b) Au_144_(SR)_60_ clusters, and their c) Ag‐ and d,e) Cu‐doped counterparts (R = C_2_H_4_Ph omitted for clarity). Au, Ag, Cu, and S atoms are depicted in yellow, grey, brown, and green, respectively.

We synthesized and characterized five distinct nanoclusters, including monometallic Au_25_(SC_2_H_4_Ph)_18_ and Au_144_(SC_2_H_4_Ph)_60_, as well as their Ag and Cu counterparts Au_25−_ *_x_* Ag*_x_* (SC_2_H_4_Ph)_18_, Au_25−_ *_x_* Cu*_x_* (SC_2_H_4_Ph)_18_, and Au_144−_ *_x_* Cu*_x_* (SC_12_H_26_)_60_. Details of their synthesis and characterization can be found in the Supplementary Information. **Scheme** **1** illustrates the structure of the synthesized clusters, highlighting variations in the structure, staple configuration, and locations of doped atoms based on reported structural studies and prior experiences.^[^ ^52^, ^53^ ^]^ Unlike Pd and Pt doping, achieving a specific number of Ag or Cu atoms in these clusters is limited because of the similarity in the electronic structures of Ag and Cu with Au (d^10^s^1^ configuration), which results in alloy clusters with a variable distribution of Cu or Ag atoms.^[^ ^54^ ^]^ Despite this, the catalytic activity of alloy clusters with a distribution of Ag/Cu dopants has been extensively studied.^[^ ^49^, ^55^ ^]^ In the current study, one to seven Ag atoms are incorporated into the surface of the metal kernel structure, while for Cu, up to 2 doping atoms are predominantly located in the staple units around the core. The characterization results by UV/Vis and MALDI‐MS corroborated the purity, as well as the composition of the nanoclusters prepared (Figures S1–S4, Supporting Information). The NCs were deposited onto a carbon black support with ≈10 wt % loading. The actual metal (Au + *M*) loading of the catalysts was quantified by thermal gravimetric analysis (TGA), which indicates a metal content between 5.5 and 7.8% (Figure S10, Supporting Information).

XPS measurements were carried out to confirm the presence of the doped metals in the NCs (Figures S5–S8, Supporting Information); the Ag 3d peak at 368.2 eV (Figure S6, Supporting Information) indicates that the incorporated Ag is neutral.^[^ ^42^ ^]^ However, the Cu 2p peak at 932.2 eV and the Cu LMM peak at 570.2 eV (Figures S7,S8, Supporting Information) indicate that Cu has an oxidation state of +1 in the bimetallic NCs.^[^ ^56^ ^]^ XPS results suggest then that while the Ag dopant incorporates in a kernel site as Ag(0), the Cu dopant is preferentially located at the staple site as Cu(I)−S, confirming previous reported studies.^[^ ^52^ ^]^

### 2.1 Electrocatalytic Activity of NCs Catalysts in CO_2_ Reduction

We have systematically investigated the influence of distinct cluster structures and metal doping on the electrocatalytic activity of the CO_2_ reduction reaction (eCO_2_R). Electrochemical assessments were conducted though 1 h chronoamperimetric experiments employing a three‐electrode H‐cell configuration, where catalyst‐modified carbon fiber paper was used as the working electrode. In this setup, a CO_2_‐saturated 0.1 M KHCO_3_ solution served as electrolyte, while Ag/AgCl and Pt coil were utilized as the reference and counter electrodes, respectively. Gas products were quantified using gas chromatography (GC), and liquid products were identified through ^1^H nuclear magnetic resonance (NMR) spectroscopy (see Electrocatalytic reaction studies on SI). Furthermore, we have explored the influence of the electrolyte cation on the electrocatalytic performance, utilizing CO_2_‐saturated 0.1 M MHCO_3_ solutions (*M* = Na, K, Cs) and catalyst‐modified glassy carbon working electrode.

### 2.2 Long Staple Protected Clusters: Au_25−_ *_x_M_x_* (SR)_18_

**Figure 1:**
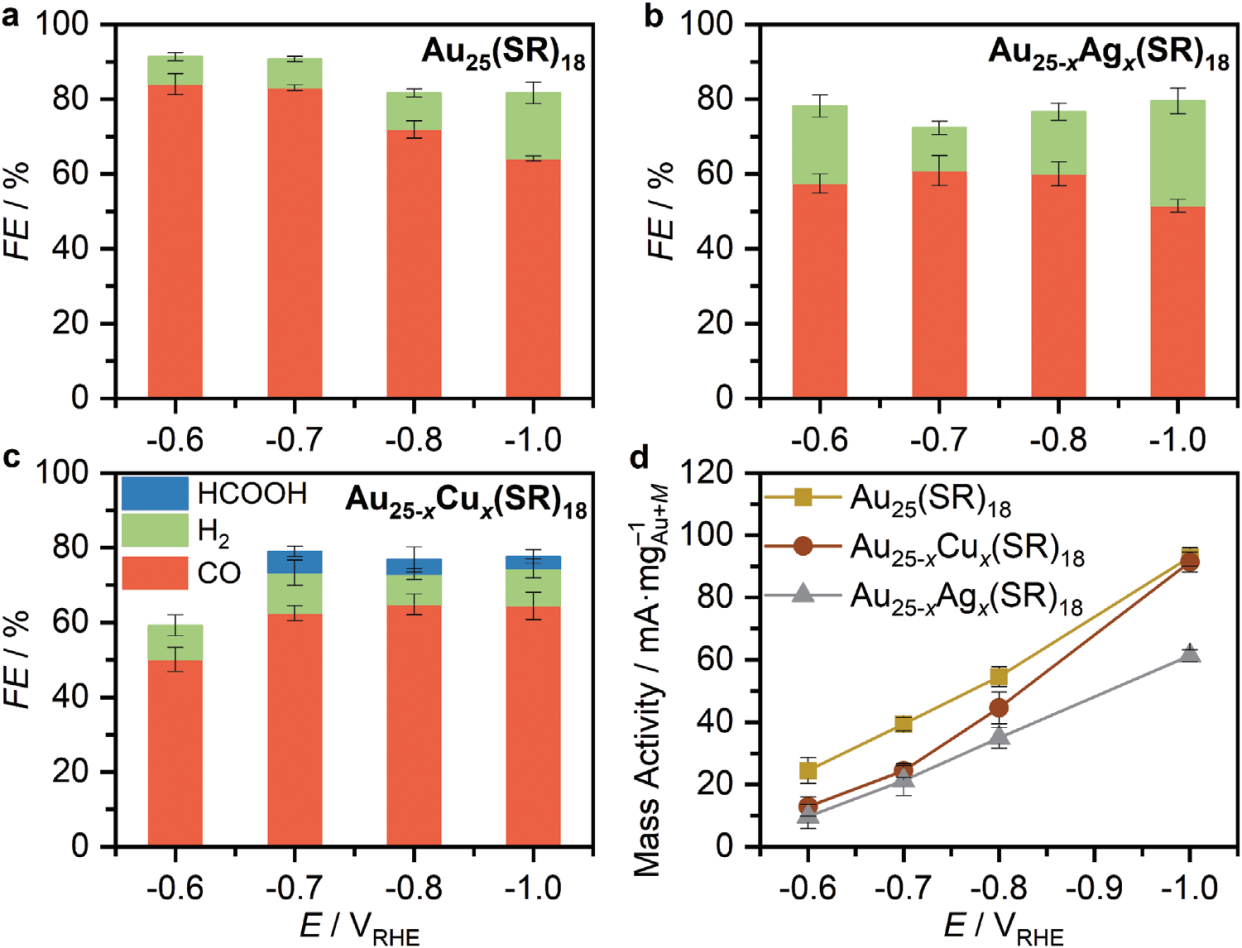
Electrocatalytic CO_2_ reduction performance of Au_25−_ *_x_M_x_* (SR)_18_ NCs. Potential‐dependent Faradaic efficiencies of a) Au_25_(SR)_18_; b) Au_25−_ *_x_* Ag*_x_* (SR)_18_; and c) Au_25−_ *_x_* Cu*_x_* (SR)_18_. d) Mass activities of the Au_25−_ *_x_M_x_* (SR)_18_ NCs. Error bars denote the standard deviations from three independent measurements.

**Figure** **1**a–c depict the electrocatalytic behavior of Au_25_‐based samples and presents the potential‐dependent Faradaic efficiencies (*FE*). CO and H_2_ were the only products detected from Au_25_(SR)_18_ and Au_25−_ *_x_* Ag*_x_* (SR)_18_ nanoclusters, aligning with previous studies on thiolate‐protected Au^[^ ^8^, ^21^, ^23^, ^31^, ^34^, ^35^, ^39^, ^57^ ^]^ and Ag^[^ ^24^, ^58^ ^]^ NCs. Interestingly, formic acid, together with CO and H_2_ have been detected using Au_25−_ *_x_* Cu*_x_* (SR)_18_, reaching a *FE*_HCOOH_ of 5.70% at −0.7 V_RHE_. Notably, while HCOOH is typically the predominant product observed for Cu^I^ hydride clusters,^[^ ^16^, ^17^, ^18^, ^45^ ^]^ its detection is uncommon for thiolate‐protected metal nanoclusters. This fact could be attributed to the absence of coadsorbed Cu*H, thereby presenting a distinct eCO_2_R reaction pathway.

In Figure 1a,b, the *FE*_CO_‐potential plots for Au_25_(SR)_18_ and Au_25−_ *_x_* Ag*_x_* (SR)_18_ show that the *FE*_CO_ decreases beyond −0.8 V_RHE_ due to increased HER. In contrast, Au_25−_ *_x_* Cu*_x_* (SR)_18_ maintains a *FE*_CO + HCOOH_ constant over a broader potential range (Figure 1c). However, it is noteworthy that the overall *FE* calculated for Au_25−_ *_x_M_x_* (SR)_18_ samples is lower than that of Au_25_(SR)_18_. For instance, at −0.7 V_RHE_, Cu doping results in a *FE*_total_ of 79% (62.5% *FE*_CO_, 5.7% *FE*_HCOOH,_ and 10% *FE*_H2_) while the undoped cluster results in a *FE*_total_ of 91% (84% *FE*_CO_ and 7% *FE*_H2_). No other gas or liquid products were detected by GC and NMR analysis, suggesting that a portion of the charge (electrons) could be playing a different role, for instance mediating the detachment of ligands. The ligand shell of these clusters may exhibit instability at high cathodic potentials, particularly in the case of Cu‐doped clusters. This is supported by NMR analysis of the electrolyte after the chronoamperometric experiments using Au_25−_ *_x_* Cu*_x_* (SR)_18_, which revealed various signals in the range from 0.5 to 2.5 ppm, attributable to alkyl chain fragments from the ligands (Figure S13, Supporting Information). Furthermore, XAFS investigations at the Au L_3_‐edge and XPS analysis, together with DFT simulations (see details below) provide robust evidence of ligand loss without affecting the metal cluster structure. Nevertheless, at −1.0 V_RHE_, Cu doping results in a *FE*_CO_ of 64.4%, similar to the undoped cluster (64.1%), a *FE*_HCOOH_ of 3.2%, and an *FE*_H2_ of 10.0%, lower than the undoped cluster (17.6%). This indicates that H_2_ evolution is significantly suppressed upon Cu doping at highly negative potentials. The mass activities (mA/mg_Au+_ *_M_*) of Au_25−_ *_x_M_x_* (SR)_18_ are presented in Figure 1d, with Au_25_(SR)_18_ having a higher mass activity than Au_25−_ *_x_M_x_* (SR)_18_ nanoclusters in the −0.6 to −0.9 V_RHE_ potential window. Interestingly, the electrocatalytic activity of Au_25−_ *_x_* Cu*_x_* (SR)_18_ matches with that of Au_25_(SR)_18_ at −1.0 V_RHE_, suggesting that Cu doping preserves the performance at highly reductive potentials, but alters selectivity in favor of CO and HCOOH products. These results demonstrate how Cu incorporation in Au_25_(SR)_18_ affects the eCO_2_R pathways, catalytic mechanism, and ligand stability.

### 2.3 Electrolyte Cation Effect

Cations from the electrolyte are crucial for eCO_2_R on coinage metal surfaces. Therefore, we investigated the impact of cations on the electrocatalytic performance of Au_25_(SR)_18_ and Au_25−_ *_x_* Cu*_x_* (SR)_18_ nanoclusters in eCO_2_R. The experiments involved applying −0.7 V_RHE_ for 1 hour in a CO_2_‐saturated 0.1 M *M*HCO_3_ (*M* = Na, K, Cs) solutions using a catalyst‐modified glassy carbon working electrode. Figure S11 (Supporting Information) shows the *FE* and mass activities at −0.7 V_RHE_ with the electrolytes containing different alkali cations. When Na^+^ was used instead of K^+^, the observed activity trend aligns with previous reports for other metal surfaces, showing an activity decrease from K^+^ to Na^+^.^[^ ^59^ ^]^ However, the use of Cs^+^ did not yield the anticipated improvement. For Au_25_(SR)_18_, the substitution of Cs^+^ for K^+^ resulted in a 25% decrease in mass activity (156.1 versus 117.2 mA mg_Au_^−1^), which is reflected in the overall decrease in *FE*. In contrast, for Au_25−_ *_x_* Cu*_x_* (SR)_18_, the mass activity and total *FE* were only slightly affected during the transition from K^+^ to Cs^+^. This finding suggests that Cs^+^ decreases eCO_2_R in the undoped cluster, whereas this effect is less pronounced after Cu incorporation. The result supports the notion that Cu doping affects the reaction pathways of eCO_2_R and/or the stability of the nanocluster.

### 2.4 Short Staple Protected Clusters: Au_144−_ *_x_M_x_* (SR)_60_

**Figure 2:**
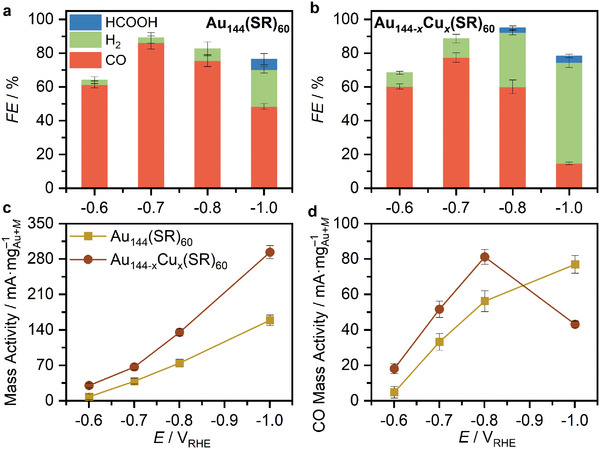
Electrocatalytic CO_2_ reduction performance of Au_144−_ *_x_M_x_* (SR)_60_ NCs. Potential‐dependent Faradaic efficiencies of a) Au_144_(SR)_60_; b) Au_144−_ *_x_* Cu*_x_* (SR)_60_. c,d) Mass activities of the Au_144−_ *_x_M_x_* (SR)_60_ NCs. Error bars denote the standard deviations from three independent measurements.

The cluster size, curvature, geometry, and staple configuration influence in eCO_2_R were studied using the Au_144−_ *_x_M_x_* (SR)_60_ nanocluster catalysts. In contrast to Au_25_(SR)_18_ clusters, which have an icosahedral Au_13_ core protected by dimeric long surface staples (−SR−Au−SR−Au−SR−), Au_144_(SR)_60_ clusters feature a hollow icosahedral Au_114_ core protected by monomeric short staples (−SR−Au−SR−) (Scheme 1). **Figure** **2** presents the potential‐dependent Faradaic efficiencies (*FE*) and mass activities for Au_144−_ *_x_M_x_* (SR)_60_ nanoclusters.

For Au_144_(SR)_60_, CO is the primary product, with a small percentage of H_2_ increasing at more reductive potentials (Figure 2a). Remarkably, undoped Au_144_(SR)_60_ produces formic acid (≈6%) at −1.0 V_RHE_ unlike undoped Au_25_(SR)_18_, where HCOOH was not detected (Figure 1a), suggesting different mechanisms leading to formic acid in both clusters. Figure 2b shows that Au_144−_ *_x_* Cu*_x_* (SR)_60_ exhibits a decrease in *FE*_CO_, an increase in *FE*_H2_, and the appearance of formic acid, at potentials more negative than −0.7 V_RHE_. Notably, *FE*_CO_ sharply decreases at −1.0 V_RHE_, contrasting Au_25−_ *_x_* Cu*_x_* (SR)_18_, where CO remains the main product at highly negative potentials (Figure 1c). Figure 2c plots the mass activities of Au_144−_ *_x_M_x_* (SR)_60_ nanoclusters; contrary to Au_25−_ *_x_M_x_* (SR)_18_ clusters, the total mass activity is higher for Au_144−_ *_x_M_x_* (SR)_60_ clusters doped with Cu. Moreover, the partial CO mass activity shows its higher value for Au_144−_ *_x_* Cu*_x_* (SR)_60_ at *U* = −0.8 V_RHE_, however, it dramatically decreases at −1.0 V_RHE_ due to an increase in H_2_ production, Figure 2d.

Our results highlight that Cu in Au_144_(SR)_60_ enhances eCO_2_R catalytic activity at *U* > −0.8 V_RHE_, while Cu in Au_25_(SR)_18_ shows lower mass activity than non‐doped cluster at *U* > −1.0 V_RHE_ and enables HCOOH production. Additionally, the comparison between the mass activities of Au_144_(SR)_60_ and Au_25_(SR)_18_ in Figure S12 (Supporting Information) indicates that the mass activity of Au_144_(SR)_60_ is higher in the potential window between −0.7 and −1.0 V_RHE_. Previous studies by Lee et al.^[^ ^39^ ^]^ analyzing Au_144_(SR)_60_, Au_38_(SR)_24_, and Au_25_(SR)_18_ indicated that the eCO_2_R activity was related to the total number of dethiolated active sites rather than the staple type. On the other hand, a recent study by Jin et al.^[^ ^40^ ^]^ indicated that eCO_2_R activity increases for smaller NCs. This suggests that the surface‐to‐volume ratio (higher for smaller clusters) and number of dealkylated active sites are the dominant factors that determine the catalytic activity. Our findings, however, indicate that the geometry of the cluster and/or the staple type (dimeric long or monomeric short) may also play a significant role in influencing activity and stability. This is particularly evident at highly negative potentials, where the Au_144_(SR)_60_ demonstrates higher activity than Au_25_(SR)_18_, despite the latter having a higher ratio of active sites for the same gold mass loading. Importantly, only Au_144_(SR)_60_ produces formic acid at −1.0 V_RHE_, underscoring the effect of cluster configuration on reaction selectivity, as seen previously for Au_38_(SR)_24_ isomers, where the differences in the core structure and staple motifs changed the selectivity.^[^ ^40^ ^]^

### 2.5 Structure Dynamic Studies

After the electrocatalytic studies, we delved into the structural stability of the cluster catalyst through X‐ray absorption fine structure (XAFS) investigations at the Au L_3_‐edge and XPS analysis. The X‐ray absorption spectroscopy (XANES) of both electrode‐supported monometallic and bimetallic clusters is shown in Figure S14 (Supporting Information). The slight changes observed in the XANES spectra are mainly related to the ligand loss after the electrocatalytic studies. The subtle shifts in the spectra, indicate a more metallic state of gold during the electrocatalytic tests. In the case of the Au_25_(SR)_18_ cluster samples, there is a noticeable variation in the intensity of the white line around 11 926 eV, attributed to changes in the occupied/unoccupied 5d states (d‐hole). These states imply charge transfer between Au and other metals and could be linked to alterations in the Au−S bond as well as the introduction of heteroatom doping. Doping with Cu induces a slight increase in intensity for Au_25_(SR)_18_ clusters, whereas such an effect is not observed for Au_144_(SR)_60_. The limited amount of copper relative to gold in the cluster hinders a comprehensive evaluation of the Cu effect, leading to a restriction of the studies to the Au L_3_‐edge.

**Figure 3:**
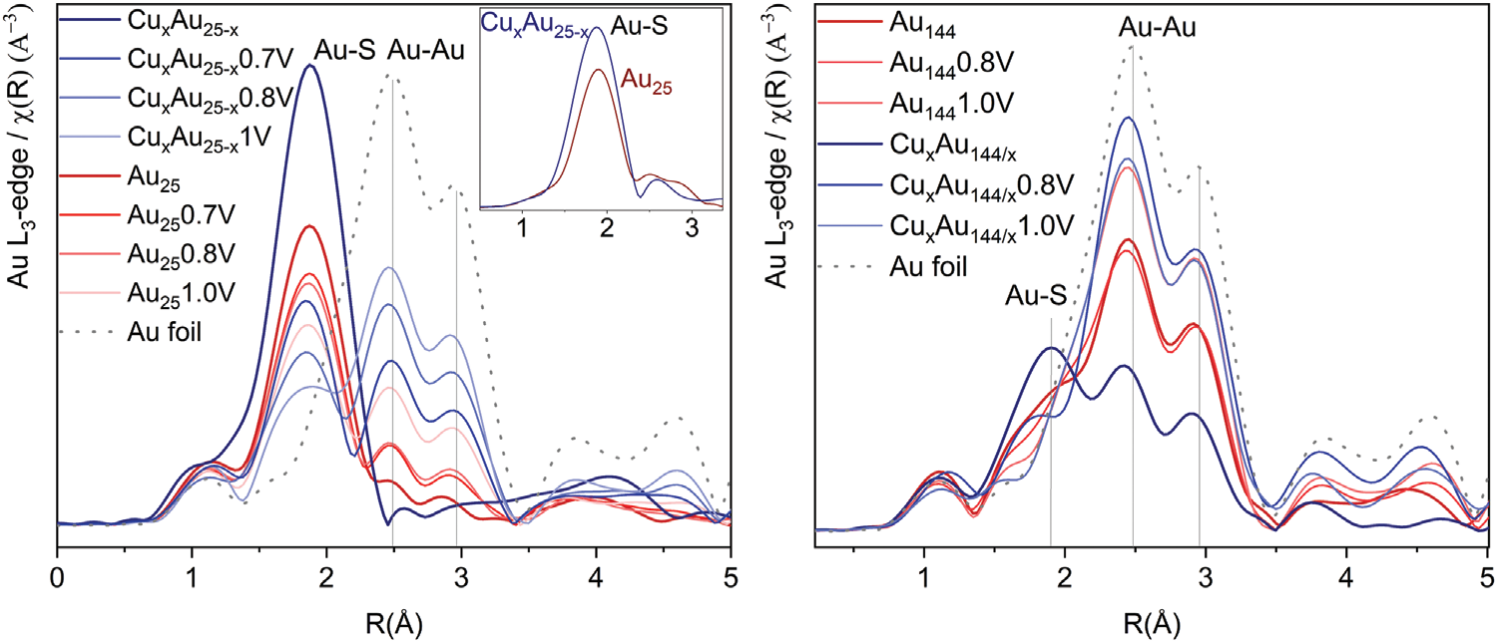
Au L_3_‐edge EXAFS results using R space figures of both monometallic and bimetallic clusters.

The stability of the main cluster structure was revealed through EXAFS (extended X‐ray absorption fine structure spectroscopy) analysis. **Figure** **3** presents the R‐space results from the Au L_3_‐edge EXAFS, along with the corresponding fitting results detailed in **Table** **1** (additional fitting information can be found in the Supporting Information, Figures S15,S16, Supporting Information). The fitting values for the fresh electrode‐supported clusters (before electrocatalytic reaction) reveal average coordination numbers (CNs) for Au−S and Au−Au that align with theoretical models and previous XAFS studies.^[^ ^60^, ^61^ ^]^

**Table 1:** Fitting results derived from the Au L_3_‐edge EXAFS data using R space for electrode‐supported clusters before (fresh) and after electrocatalytic reaction at various applied potentials.

		Au−S		Au−Au	
		CN	R (Å)	CN	R (Å)
Au_25_	fresh	1.79 ± 0.28	2.30 ± 0.03	1.04 ± 0.63	2.77 ± 0.13
	−0.7 V	1.52 ± 0.28	2.31 ± 0.04	2.08 ± 0.75	2.82 ± 0.07
	−0.8 V	1.47 ± 0.25	2.30 ± 0.04	2.25 ± 0.66	2.82 ± 0.06
	−1.0 V	1.21 ± 0.25	2.31 ± 0.04	3.99 ± 0.82	2.84 ± 0.04
Cu_x_Au_25‐x_	fresh	2.83 ± 0.72	2.29 ± 0.05	1.16 ± 1.54	2.53 ± 0.29
	−0.7 V	1.36 ± 0.57	2.30 ± 0.12	4.56 ± 1.86	2.84 ± 0.07
	−0.8 V	1.04 ± 0.58	2.31 ± 0.11	6.19 ± 1.97	2.85 ± 0.04
	−1.0 V	0.72 ± 0.39	2.30 ± 0.09	7.64 ± 1.52	2.85 ± 0.09
Au_144_	fresh	0.55 ± 0.85	2.32 ± 0.33	7.98 ± 1.81	2.84 ± 0.07
	−0.8 V	0.45 ± 0.47	2.33 ± 0.22	7.79 ± 1.23	2.84 ± 0.04
	−1.0 V	0.16 ± 0.47	2.33 ± 0.63	10.15 ± 1.90	2.85 ± 0.03
Cu_x_Au_2144‐x_	fresh	0.95 ± 0.30	2.32 ± 0.07	4.45 ± 1.02	2.83 ± 0.04
	−0.8 V	0.15 ± 0.47	1.72 ± 0.67	10.88 ± 1.86	2.85 ± 0.07
	−1.0 V	0.17 ± 0.94	2.32 ± 1.19	10.31 ± 2.00	2.85 ± 0.06

Analysis of the clusters after the different electrocatalytic tests indicates substantial changes, in particular a decrease in Au−S CN and a slight increase in Au−Au CN. The decrease in Au−S denotes the loss of ligands, at the staple structure, suggesting the breaking of Au−S bonds during electrochemical reactions. This loss is more pronounced in Cu‐doped clusters in both cases, consistent with the observed catalytic activity and theoretical simulations (see below). The increase in the average Au−Au CN is consistent with the loss of Au−S bonds and, in all cases, remains below CN = 12, which would correspond to Au foil structure, indicating an increase in cluster size. Conclusively, Au L_3_‐edge XAFS data indicates that while the structure of clusters is maintained during catalytic reaction, the ligand shell suffers instability during eCO_2_R reactions.

S K‐edge measurements were performed to further investigate the stability of the ligand shell and evolution after reaction. However, we encountered a significant difficulty: the presence of additional S species originating from the sulfonate groups in the Nafion polymer commonly used in electrode preparation for these electrocatalytic experiments. This sulfonate group produced a strong doublet around 2480 eV, dominating the S spectrum of the sample and masking the signal from the thiolate ligands (Figure S17, Supporting Information). To avoid the issues encountered in XAS measurements, electrodes containing Cu‐doped cluster, Au_25−_ *_x_* Cu*_x_* (SR)_18_ sample were prepared in the absence of Nafion and XPS analysis was performed before and after electrocatalytic testing. XPS reveals that the S / Au ratio decreased after 1 h chronoamperometry experiments at −0.7 V_RHE_ (Figure S9 and Table S1, Supporting Information). We have quantified 20.8% of ligands lost after electrocatalytic experiments, in agreement with our previous results and those of the state‐of‐the‐art.^[^ ^39^ ^]^ These results support our conclusions from NMR and Au L_3_‐edge EXAFS data after catalysis, indicating the detachment of ligands (especially in Cu‐doped clusters).

### 2.6 Correlation Structure Dynamics to Electrochemical Activity: DFT Simulations

#### 2.6.1 Larger Staples: Au_25−_ *_x_M_x_* (SR)_18_ Dopants Incorporation and Ligands Stability

Then we modeled bimetallic clusters using DFT simulations by incorporating *M_x_* dopants on optimized Au_25_(SR)_18_ structure kernel (k) and staple (s) positions, resulting in Au_25−_ *_x_M_x_* (SR)_18_ structures (*M* = Cu, Ag; *x* = 1k, 1s, 2k, 2s, 2k,s). The energies of a single *M* incorporation in kernel positions are lower than in staple (ΔΔ*E_M_* = −0.17 and −0.33 eV, for Cu and Ag respectively), suggesting their higher stability in agreement with previous DFT results.^[^ ^41^ ^]^ However, XAFS analysis has shown that Cu incorporates in staples,^[^ ^52^ ^]^ thus its presence in both staples and kernel positions cannot be ruled out first because they are close in energy and second because staples are more kinetically accessible. The energies of a second *M* incorporation are isoenergetic for both the closest (Figure S18, Supporting Information) and the farthest positions (Table S2, Supporting Information) to the first *M* incorporated. This indicates that there is no preference for *M* to segregate within the cluster structure. Therefore, Cu can be present in close kernel and staple positions, while structures with Ag in staples are less favorable according to the computed energies and previous experimental literature.^[^ ^62^ ^]^

**Figure 4:**
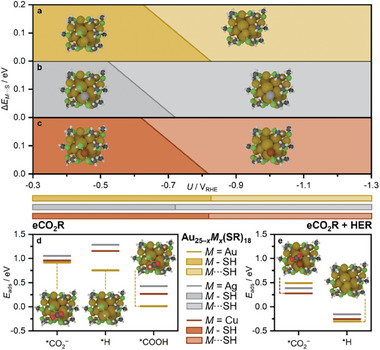
a, b, c) Phase diagrams for the ligand stability of Au_25−_ *_x_M_x_* (SR)_18_ under applied potential (*U*) for (a) *M_x_* = Au, (b) *M_x_* = Ag_1s_, and (c) *M_x_* = Cu_1s_. Dethiolation energies (Δ*E_M_*_···S_) are computed as a single *M*···SH dissociation on Au_25−_ *_x_M_x_* (SR)_17_(SH) to form H_2_S and Au_25−_ *_x_M_x_* (SR)_17_. d, e) Adsorption energies of the intermediates leading to CO (*CO_2_^−^ and *COOH), and H_2_ (*H) products on (d) Au_25−_ *_x_M_x_* (SR)_17_(SH) staple site and (e) Au_25−_ *_x_M_x_* (SR)_17_ kernel site (*M_x_* = Au, Cu_1s_, Ag_1s_). *CO_2_^−^ and *COOH adsorption energies are computed at *U* = −0.7 V_RHE_, and *H adsorption energies are at *U* = 0.0 V_RHE_. For each intermediate the optimized structure giving the lowest energy is shown.

We further assessed the stability of terminal −SR ligands (which link to the gold core) and central −SR ligands (which link to the two staple gold atoms) by considering the S···R bond dealkylation (Δ*E_M_*_‐S···R_) resulting in Au_25−_ *_x_M_x_* (SR)_17_(SH) cluster, and further ligand *M*···SH dethiolation (Δ*E_M_*_···S_) to Au_25−_ *_x_M_x_* (SR)_17_ (see Ligand elimination energies SI). For Au_25_(SR)_18_ the dealkylation is favored for both terminal and central ligands (Δ*E_M_*_‐S···R_ = −0.72 and −0.75 eV, respectively) at *U* = 0.0 V_RHE_. However, at this potential, further dethiolation is endothermic with higher values for central ligands (Δ*E_M_*_···S_ = 1.38 eV) than terminal ones (Δ*E_M_*_···S_ = 0.83 eV), Table S3 (Supporting Information). Switching to more reductive potentials a single terminal −SH group per staple can be decoordinated (Δ*E_M_*_···S_ = 0.03 eV at *U* = −0.8 V_RHE_) but a second dethiolation is unfeasible (Δ*E_M_*_···S_ = 0.90 eV), Table S4 (Supporting Information). Ligand stability is unaffected by *M* dopants except for *M_x_* = Cu_1k_ which increases the terminal *M*···SH elimination energy (ΔΔ*E_M_*_···S_ = 0.40 eV), Table S3 (Supporting Information). However, for *M_x_* = Au, Cu_1s_, and Ag_1s_ the dethiolation energies plotted against the applied potential (*U*) show that at −0.8 V_RHE_ the terminal ligand decoordination starts to be favored thus compromising ligands stability, **Figure** **4**a–c. This stability window correlates with the decrease in *FE*_CO_ starting at the same potential (Figure 1a), suggesting that if ligands stay on the cluster, they favor CO generation by blocking the kernel and limiting reactivity to the more active staple sites. Interestingly, for *M_x_* = Cu_1k_ the terminal ligands are stable down to *U* = −1.0 V_RHE_, thus Cu incorporation on the kernel would enable the tunning of reactivity by limiting it on staples even at these reductive potentials (Figure S19, Supporting Information). Moreover, the ligand elimination modifies the electronic properties of the cluster as indicated by Bader charges (Table S5, Supporting Information) analysis for *M* atoms, which can also affect the reactivity.

#### 2.6.2 Au_25−_ *_x_M_x_* (SR)_18_ Reactivity

We then investigated the mechanisms leading to the detected products (CO, H_2_, and HCOOH) on Au_25−_ *_x_M_x_* (SR)_18_ clusters. The most widely accepted eCO_2_R route for CO production starts with CO_2_ coordinated and activated via electron transfer followed by protonation to *COOH, a proton‐coupled electron transfer to produce water and *CO, and ends with CO desorption.^[^ ^63^, ^64^ ^]^ Alternatively, the competing HER mechanism starts with *H adsorption and further reduction to hydride (*H^−δ^).^[^ ^65^ ^]^ Then it can proceed to react with a proton in the electrolyte to produce H_2_ or also interact with solvated CO_2_ which leads to HCOOH, both following a Heyrovsky mechanism.^[^ ^66^ ^]^ Also formic acid can be produced through bidentate h_O,O_ *OCO*^−^ adsorption and further protonation to *HCOO,^[^ ^67^ ^]^ or through the generation of a thiocarbonate intermediate (−S−CO_2_^−^).^[^ ^68^ ^]^ The stability of key precursors of the described mechanisms to CO and H_2_ (*CO_2_^−^ and *H) was first assessed on staple sites, their adsorption energies (*E*_ads_) being endothermic for all *M*, Figure 4d. While *CO_2_^−^ adsorption is barely affected by the *M* dopant, more positive *H adsorption values are obtained on *M_x_* = Cu_1s_, Ag_1s_ (*E*_ads_ = 1.16 and 1.28 eV, respectively) than on *M_x_* = Au (*E*_ads_ = 0.75 eV), suggesting that HER is hindered on more electropositive Cu and Ag atoms.^[^ ^69^ ^]^ This is supported by Bader charge analysis (Table S5, Supporting Information) which shows that Cu and Ag atoms in staples are more polarized than Au due to ligands electrophilicity (*q*_B_ = 0.42, 0.32, and 0.08 |e^−^|, respectively).^[^ ^45^ ^]^

The hindered HER mechanism on staple sites agrees with the high *FE*_CO_ experimentally observed *U* = −0.7 V_RHE_ (Figure 1), suggesting that the *CO_2_^−^ adsorption is produced despite the obtained energy penalties.^[^ ^70^ ^]^ Moreover, a significant difference was observed in adsorption energies of *COOH intermediate, being thermoneutral for *M_x_* = Au but endothermic for *M_x_* = Cu_1s_, Ag_1s_ at *U* = −0.7 V_RHE_, Figure 4d. Therefore, on Cu‐doped NCs the energy penalty for *COOH adsorption (0.27 eV) is higher than Δ*E_M_*_···S_ (0.10 eV) thus a more favored ligand sacrifice consumes part of the eCO_2_R current, while the more stable *COOH (0.01 eV) on non‐doped Au_25_(SR)_18_ explains the higher *FE*_CO_ observed.

Complementary we assessed the stability of *CO_2_^−^ and *H precursors on kernel sites, which become accessible after dethiolation at potentials more reductive than −0.8 V_RHE_. Both reactants bind stronger on kernel than on staple sites, and *H adsorption energy decreases more significantly (ΔΔ*E* = −1.44 – −1.06 eV) than *CO_2_^−^ (ΔΔ*E* = −0.68 – −0.43 eV), Figure 4d. The lower Bader charges of these kernel atoms (*q*_B_ = −0.15 – −0.08 |e^−^|) compared to staple *M* also suggest a more favored *H adsorption, Table S5 (Supporting Information). Moreover, for all studied clusters *H adsorption was endothermic on staple and exothermic on kernel sites (Table S6, Supporting Information), and the difference between them was larger for charged clusters (*q* = −1) following the superatom electronic theory.^[^ ^71^, ^72^ ^]^ Thus, the promoted HER on kernel sites indicates that ligand elimination compromises the clusters’ eCO_2_R performance, in agreement with the lower *FE*_CO_ observed in experiments at potentials where dethiolation is expected (*U* = −0.8 V_RHE_).

It has been demonstrated that the endothermic activation of CO_2_ on inert metal surfaces such as Au, Ag, and Cu is enabled by cations.^[^ ^70^, ^73^ ^]^ Thus, the role of cations on the stability of the key precursors on staple and kernel sites was assessed using partially solvated cations with *n* H_2_O (K^+^‐*n*H_2_O and Cs^+^‐*n*H_2_O; *n* = 3, 5) for *M_x_* = Au, Cu_1s_. On staple sites, the decrease of energy penalties for *CO_2_^−^ (ΔΔ*E* = −2.06 and −1.44 eV for *M_x_* = Au and Cu_1s_, respectively) (ΔΔ*E* = −0.89 eV) contrasts with *H adsorption energies which are not affected by cations, Figure S20a,b (Supporting Information). However, on kernel sites *CO_2_^−^ adsorption is less promoted (ΔΔ*E* = −0.54 and −0.52 eV for K^+^ and Cs^+^) and *H adsorption is only enhanced by Cs^+ [^ ^74^ ^]^ (ΔΔ*E* = −0.20 eV) Figure S20c (Supporting Information). These results not only confirm that cations enable CO_2_ binding on staple sites, but also suggest that Cs^+^ enhances HER on kernel sites which agrees with the experimental observations for Au_25_(SR)_18_ (Figure S11, Supporting Information).

The full reaction profiles for the different *M* dopants on staple sites were computed for Au_25−_ *_x_M_x_* (SR)_16_(SH)_2_ and also for charged Au_25−_ *_x_* (SR)_16_(SH)_2_^−^ clusters (*M_x_* = Au, Cu_1s_, Cu_1k_, Cu_2k,s_, Ag_1s_, Ag_1k_, and Ag_2k,s_), Figure S21 (Supporting Information). A more favored pathway to CO is observed on *M* = Au in agreement with the experimentally measured activities (Figure 1d). The same reaction pathways were computed on kernel sites for Au_25−_ *_x_* (SR)_16_(SH) and Au_25−_ *_x_* (SR)_16_(SH)^−^, showing high energy penalties on CO desorption step (Δ*E* = 0.67 eV) despite they bind better *CO_2_^−^ than staple sites, Figure S22 (Supporting Information). Moreover, the profiles to HCOOH through thiocarbonate on *M_x_* = Cu_1s_ and Cu_2k,s_ were also computed, Figure S23 (Supporting Information). The initial CO_2_ adsorption on the −SH ligand to form −S−CO_2_^−^ displaces the H to any of the available kernel sites (Figure S23e, Supporting Information) and has an energy penalty (Δ*E* = 0.40 – 0.46 eV), but the rest of the reaction is downhill. In summary, Cu incorporation in Au_25_(SR)_18_ enables HCOOH formation but at the cost of a partial reduction in eCO_2_R performance due to: i) the significant ligand instability at potentials rounding −0.7 V_RHE_, exposing more kernel sites which tend to favor HER; and ii) the higher energy barrier for *COOH adsorption, resulting in a preference for the ligands to detach rather than facilitate the reaction.

#### 2.6.3 Shorter Staples: Au_144−_ *_x_M_x_* (SR)_60_ Dopants Incorporation and Ligands Stability

**Figure 5:**
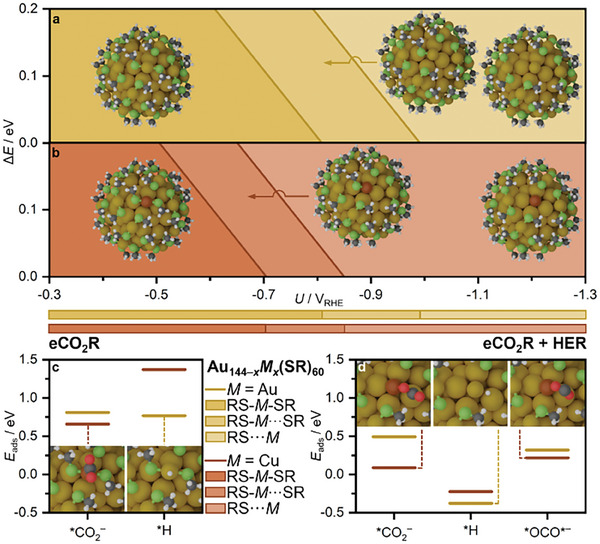
a,b) Phase diagrams of Au_144−_ *_x_M_x_* (SR)_60_ ligand stability under applied potential (*U*) for (a) *M_x_* = Au and b) *M_x_* = Cu_1s_. Energies of the first and second dethiolations (Δ*E_M_*_···S_) in the same staple are computed as RS−*M*···SR and RS···*M* bond dissociations to HS−CH_3_, respectively. c,d) Adsorption energies of the first intermediates leading to CO, H_2_, and HCOOH products on (c) Au_144−_ *_x_M_x_* (SR)_60_ staple site and (d) kernel site (*M_x_* = Au and Cu_1s_). *CO_2_^−^ and *OCO*^−^ adsorption energies are computed at *U* = −0.7 V_RHE_, and *H adsorption energies are at *U* = 0.0 V_RHE_. For each intermediate the optimized structure giving the lowest energy is shown.

The incorporation of one and two *M* atoms in Au_144_(SR)_60_ was studied using the same positions as for Au_25_(SR)_18_ (*M* = Cu, Ag; *x* = 1k, 1s, 2k, 2s, 2k,s). In contrast with these smaller clusters where dopants are more stable on kernel positions, the energies of Cu incorporation in staple and kernel sites of Au_144−_ *_x_M_x_* (SR)_60_ are isoenergetic (Δ*E_M_* = −0.11 – −0.07 eV), Figure S24 (Supporting Information). Also, the Cu effect on ligand elimination energy is less significative for Au_143_Cu_1k_(SR)_60_ than for Au_24_Cu_1k_(SR)_18_ (Δ*E* = 1.12 and 1.38 eV at *U* = 0.0 V_RHE_, respectively), and the first and second dethiolations in the same staple show similar energies, Table S7 (Supporting Information). These two consecutive dethiolation energies (RS−*M*···SR and RS···*M*, respectively) were plotted against applied potential (*U*) for *M* = Au and Cu, **Figure** **5**a,b. The phase diagrams show that at potentials more reductive than −0.8 V_RHE_ one ligand per staple is eliminated (both for *M* = Cu), leading to partially or completely dethiolated clusters

#### 2.6.4 Au_144−_ *_x_* M*_x_* (SR)_60_ Reactivity

The adsorption energies of the precursors leading to eCO_2_R and HER were evaluated both on staple and kernel sites, Figure 5c,d, and Figure S25a,b (Supporting Information). With Cu in staples lower *CO_2_^−^ and significantly higher *H adsorption energies are obtained compared with *M* = Au, in agreement with the better activity observed in the experiments (Figure 2c,d). On kernel sites, the lower adsorption energies of both precursors (especially *H) suggest a preferential reactivity on kernel when it becomes accessible after staple first dethiolation at *U* = −0.8 and −0.7 V_RHE_ for *M* = Au and Cu, respectively. This agrees with the experiments on Au_144−_ *_x_M_x_* (SR)_60_ clusters (Figure 2a,b) where *FE*_CO_ decays earlier and more significantly than on Au_25_(SR)_18_ clusters, also happening at the potentials where ligand elimination is expected. Moreover, the sharp decrease in *FE*_CO_ observed for *M* = Cu at *U* = −1.0 V_RHE_ follows the almost total dethiolation of the cluster which leads to sintering to larger particles.

**Figure 6:**
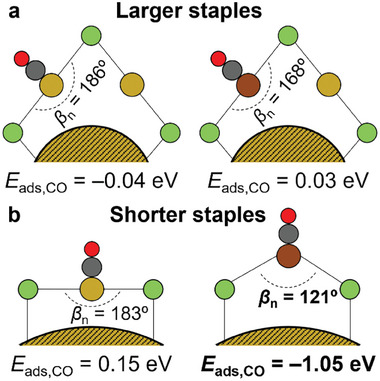
Schematic representation of staples bite angle (βn) modification during CO adsorption for a) Au_25−_ *_x_M_x_* (SR)_18_ and b) Au_144−x_M_x_(SR)_60_ staples (*M_x_* = Au and Cu_1s_). *CO adsorption energies shown are at *U* = −0.7 V_RHE_.

The energy profiles of eCO_2_R to CO were evaluated for Au_144−_ *_x_M_x_* (SR)_60_ and Au_144−_ *_x_M_x_* (SR)_58_(SH)_2_ clusters on staple sites (RS−*M−*SR and HS−*M−*SH, respectively), Figure S26 (Supporting Information). They show that Cu in staple sites not only favors *CO_2_^−^ adsorption energy, but also stabilizes *CO intermediate with respect to Au (ΔΔ*E* = −0.9 eV). This lower *CO adsorption energy can be explained by the higher capacity of shorter staples (−SR−Cu−SR−) to reduce the bite angle (*β*_n_) from 182° – 183° down to 121° (**Figure** **6**), while (−SR−Au−SR−) staples and larger staples (−SR−*M−*SR−*M−*SR−) with higher distortion energies are stiffer (Table S8, Supporting Information). The difference in staples flexibility can be explained by the higher sphericity, measured by isoperimetric quotient (IQ)^[^ ^75^ ^]^ of Au_144_(SR)_60_ rhombicosidodecahedra‐like kernel^[^ ^50^, ^51^ ^]^ compared with icosahedral^[^ ^76^ ^]^Au_25_(SR)_18_ (IQ = 0.9357 and 0.8288, respectively^[^ ^77^ ^]^). On the other hand, the energy profiles on kernel sites for Au_144−_ *_x_M_x_* (SR)_59_ and Au_144−_ *_x_M_x_* (SR)_58_ clusters show a high energy penalty of CO desorption step (ΔΔ*E* = 0.71 – 0.97 eV), Figure S25c,d (Supporting Information). Thus, eCO_2_R to CO will be favored at potentials where kernel sites are blocked by ligands, with an enhanced performance if Cu is incorporated in staples.

Moreover, the HCOOH production through bidentate *OCO*^−^ was investigated instead of the thiocarbonate route, as the product is also detected on non‐doped Au_144_(SR)_60_ at potentials where high dethiolation is expected. A more favored *OCO*^−^ adsorption is observed on Au_144−_ *_x_M_x_* (SR)_60_ than on Au_25−_ *_x_M_x_* (SR)_18_ kernel (Δ*E*_ads_ = −0.6 – −0.5 eV), Figure 5d. Moreover, the energy profile to HCOOH through *OCO*^−^ was more favored for *M* = Cu after a single ligand elimination (Figure S25e, Supporting Information) and even more favored on completely dethiolated staples (Figure S25f, Supporting Information), in good agreement with the experiments (Figure 2a,b). These results confirm that HCOOH can be produced on ligand‐depleted Au_144−_ *_x_M_x_* (SR)_60_ clusters, while on staples the thiocarbonate route is ruled out as no stable −S−CO_2_^−^ structures were found.

## 3 Conclusion

In summary, our study explored the intricate interplay between ligand configuration and eCO_2_R activity, focusing on thiolate‐protected Au_25_(SR)_18_ and Au_144_(SR)_60_, with different staple motifs and introduced dopants (Cu and Ag). The comprehensive analysis, combining experimental characterizations, electrocatalytic activity measurements, and DFT simulations, highlighted the central role of ligand stability in determining the activity and selectivity of eCO_2_R. We reveal how bimetallic clusters can be used to tune their microenvironment and the stability of key reaction intermediates, which leads to improving their efficiency as eCO_2_R catalysts. Our experiments and mechanistic studies show that Cu in Au_25_(SR)_18_ can extend the optime selectivity to higher reductive potentials (*U* = −1.0 V_RHE_) and enable HCOOH production, while Cu in Au_144_(SR)_60_ can enhance the CO activity at *U* > −0.8 V_RHE_. However, in the case of Cu‐doped Au_25_(SR)_18_ the energy cost associated with *COOH adsorption outweighs that of ligand dissociation, leading to a preferential sacrifice of ligands and partial reduction in eCO_2_R efficiency. Ligand stability under reaction conditions is influenced by their configuration, with long staples within Au_25−_ *_x_M_x_* (SR)_18_ exhibiting a single dethiolation per staple site under highly reductive potentials and short staples within Au_144−_ *_x_M_x_* (SR)_60_ showing two consecutive ligand decoordination steps leading to complete dethiolation. Furthermore, the presence or absence of thiolate ligands influences the site and configuration of absorbed reactants (at the staple or the surface core structure of the metal clusters), thereby resulting in different product distribution. Understanding these fundamental insights can contribute to an optime design of atomically precise Au NCs, paving the way for the utilization of bimetallic clusters for highly efficient eCO_2_R.

## Conflict of Interest

The authors declare no conflict of interest.

## Supporting information



Supporting Information

## Data Availability

The data that support the findings of this study are openly available in [ioChem‐BD] at [https://www.iochem‐bd.org/handle/10/370594], reference number [10060421].
