# Supplementary material for: Structural Evolution of Stapes Controls the Electrochemical CO2 Reduction on Bimetallic Cu‐doped Gold Nanoclusters
Source: Small. 2024 Dec 2;21(2):2408531. doi: 10.1002/smll.202408531 (PMC11735902; doi:10.1002/smll.202408531)
Supplement: Supplementary file 1 — Supporting Information [file SMLL-21-2408531-s001.docx]

***Supporting Information***

Structural Evolution of Stapes Controls the Electrochemical CO_2_ Reduction on Bimetallic Cu-doped Gold Nanoclusters

Enric Ibáñez-Alé,^‡ [a, b]^ Jiajun Hu,^‡ [c]^ Josep Albero,^[c]^ Laura Simonelli,^[d]^ Carlo Marini,^[d]^ Núria López,*^[a]^ Noelia Barrabés,*^[e]^ Hermenegildo García,*^[c]^ Sara Goberna-Ferrón,*^[c]^

[a] E. Ibáñez-Alé, Prof. N.López
Institute of Chemical Research of Catalonia, (ICIQ-CERCA), The Barcelona Institute of Science and Technology (BIST)
Av. Països Catalans 16, 43007 Tarragona, Spain
nlopez@iciq.es

[b] E. Ibáñez-Alé
Universitat Rovira i Virgili
Avinguda Catalunya, 35, 43002 Tarragona, Spain

[c] Dr. J. Hu, Dr. J Albero, Prof. H.García, Dr. S Goberna-Ferrón
Institution Instituto Universitario de Tecnología Química (CSIC-UPV), Universitat Politècnica de València
Avda. De los Naranjos s/n, Valencia 46022, Spain

[hgarcia@itq.upv.es](mailto:hgarcia@itq.upv.es), sgobfer@itq.upv.es

[d] Dr. L. Simonelli, Dr. C. Marini
ALBA Synchrotron Light Facility
Carrer de la Llum 2-26, 08290, Cerdanyola del Valles, Barcelona, Spain

[e] Dr. N. Barrabés
Institute of Materials Chemistry, Technische Universität Wien
Getreidemarkt 9/BC/01, Vienna 1060, Austria.

[^‡^] These authors contributed equally

**Methodology**

**Nanoclusters Synthesis**

The Au_25_(SR)_18_ and Au_144_(SR)_60_ (SR = 2-phenylethanethiol (2-PET)) clusters were prepared according to the previous methods.^[1,2]^

***Au_25–x_Ag_x_(SR)_18_*** NCs were synthesized following a modified protocol from Gottlieb *et al*.^[3]^ The reaction was carried out under an argon atmosphere. Briefly, 0.609 mmol of HAuCl_4_.3H_2_O (>99.9% metals basis, Aldrich) were put in a round-bottom flask by taking 2.674 mL of an aqueous (89.752 g / L) HAuCl_4_.3H_2_O stock solution and removing the water by rotatory evaporation. Afterward, 0.028 g (0.126 mmol) of AgCF_3_COO (>99.9% metals basis, Aldrich) was added. After adding 0.465 (0.859 mmol) g of TOAB (≥98%, Aldrich) as a phase transfer agent and 45 mL of THF (tetrahydrofuran) as the solvent, the resulting red solution was stirred for 15 minutes. Then, 0.500 mL (3.73 mmol) of 2-PET (2-Phenylethanethiol, 98%, Aldrich) ligand was added, which led to a slow color change towards yellow. The solution was then stirred again for 15 minutes. As a reducing agent, 0.279 g (7.380 mmol) of NaBH_4_ (sodium borohydride, Aldrich) were quickly dissolved in 15 ml of ice-cold ultrapure water and immediately added to the yellow solution, which then became dark brown and produced bubbles for some seconds. The mixture was stirred for five hours under an argon atmosphere. Afterward, the solution was centrifuged to remove the white solid (TOAB, NaBH_4,_ or by-products). Then, the solvent was removed by rotatory evaporation. The remaining orange oily product was washed with methanol three times by centrifugation. Clusters were then extracted with DCM (dichloromethane) and the solvent was removed by rotatory evaporation. Doped clusters were separated from undoped clusters by extraction with ACN (acetonitrile); however, the solid completely dissolved in ACN indicating that doping was successful. Then, the dark pink solution was transferred to a flask and evaporated. Finally, the clusters were separated by Size Exclusion Chromatography (SEC), and their purity was evaluated by UV-vis spectroscopy and MALDI mass spectrometry.

*Au_25–x_Cu_x_(SR)_18_* NCs were synthesized following a protocol from Negishi *et al*.^[4]^ with minor modifications. Briefly, 1.452 mmol of HAuCl_4_.3H_2_O were put in a round-bottom flask by taking 7.580 mL of an aqueous (37.660 g / L) HAuCl_4_.3H_2_O stock solution and removing the water by rotatory evaporation. Then, 0.063 g (0.369 mmol) of CuCl_2_·2H_2_O (>99.9% metals basis, Aldrich) and 1.084 g (1.983 mmol) of TOAB were dissolved in 150 mL of methanol and added to the flask. The resulting red solution was stirred for 15 minutes. Then, 2.982 mL (21.600 mmol) of 2-PET was added to the solution. The solution was stirred at room temperature for 15 min. Then 0.686 g (18.000 mmol) of NaBH_4_ dissolved in the minimum amount of ice-cold ultrapure water was rapidly added to the solution, which immediately changed from a cloudy grey-brownish solution to a cloudy black solution. The solution was stirred at room temperature for 3 h. Over time the solution decolored and insoluble black precipitate floated in the clear solution. The solid was separated by centrifugation and washed four times with methanol to remove thiol and other by-products. Acetonitrile was then added to the precipitate, and the products that had dissolved in ACN were collected. Then, the solution was transferred to a flask and evaporated to obtain a reddish-brown product. Finally, the clusters were separated SEC, and their purity was evaluated by UV-Vis spectroscopy and MALDI mass spectrometry.

*Au_144–x_Cu_x_(SR)_144_* NCs were synthesized following a protocol from Dharmaratne *et al*.^[5]^, except that 1-dodecanethiol was used as the ligand instead of 1-hexanethiol. Briefly, 0.130 mmol of HAuCl_4_.3H_2_O were put in a round-bottom flask by taking 0.679 mL of an aqueous (37.660 g / L) HAuCl_4_.3H_2_O stock solution and removing the water by rotatory evaporation. Then, 0.004 g (0.026 mmol) of CuCl_2_·2H_2_O and 0.235 g (0.468 mmol) of TOAB were dissolved in 25 mL of ethanol and added to the flask, resulting in a red coloring solution. After 15 min of stirring, 0.112 mL (0.468 mmol) of 1-dodecanethiol (≥98%, Aldrich) was added and the solution was stirred for 1h. Then, 0.378 g (10.000 mmol) of NaBH_4_ dissolved in the minimum amount of ice-cold ultrapure water was rapidly added to the solution, which immediately changed from a cloudy whitish solution to a colorless solution with an insoluble brown precipitate. The mixture was then stirred for 2 h, after which the precipitate was separated by centrifugation and washed three times with methanol to remove thiol and other by-products. The final product was then extracted by tetrahydrofuran (THF), the clusters were separated by SEC, and their purity was evaluated by UV-Vis spectroscopy and MALDI mass spectrometry.

**Cluster Characterization (MALDI-MS and UV-Vis)**

The UV−vis absorption spectra of clusters dissolved in THF were recorded with a Perkin Elmer Lambda 750 UV-vis spectrometer.

MALDI mass spectrometry measurements of Au_25–_*_x_M_x_*(SR)_18_ NCs were performed in the proteomics facility of SCSIE University of Valencia (Spain) using a TIMS-TOFF Flex (Bruker) in MALDI operation, in reflector positive mode at 200-1000 m/z and 1000-10000 m/z rang and a laser intensity of 30-40 % and 40-60 %. Samples were dissolved in DCM and diluted in the DCTB matrix (10mg/mL DCTB (*trans*-2-[3-(4-*tert*-butylphenyl)-2-methyl-2-propenylidene]malononitrile, Aldrich) in 70% ACN, 0.1% TFA (trifluoroacetic acid)) and were then spotted and air-dried. MALDI mass spectrometry measurement of Au_144_(SR)_60_ NC was performed on a Shimadzu MALDI/TOF/RTOF 7090 mass spectrometer (Shimadzu Kratos Analytical) in positive linear mode. Ionization was achieved with a Nd:YAG laser with a wavelength of 355 nm. The sample was dissolved in toluene and diluted in a DCTB matrix and was then spotted and air-dried.

UV-vis spectroscopy (**Fig. S1**) and matrix-assisted laser desorption/ionization mass spectroscopy (MALDI-MS) (**Fig. S2-4**) were used to confirm the purity of the clusters. The UV-vis spectrum of Au_25_(SR)_18_ (**Fig. S1a**) shows the distinct absorption bands at 1.8, 2.8, and 3.1 eV.^[6]^ *M* doping clearly modiﬁes the optical properties of the Au_25_(SR)_18_ cluster; the spectrum of Au_25–x_Ag_x_(SR)_18_ shows features at about 1.8, 2.4, and 2.6 eV in agreement with experimentally reported spectra,^[7,8]^ and the spectrum of Au_25–x_Cu_x_(SR)_18_ appears broadened and slightly shifted to a lower energy relative to Au_25_(SR)_18_ (this shift has been observed for Au_∼24_Cu_∼1_(SR)_18_ clusters^[4]^ while broadening seems to appear by increasing Cu doping^[8]^ ). The UV-vis spectrum of Au_144_(SR)_60_ (**Fig. S1b**) exhibits the characteristic absorption bands at ∼2.4 eV and ∼1.8 eV,^[1,9]^ while the spectrum of Au_144–x_Cu_x_(SR)_60_ shows the surface plasmon-like broad peak at ∼2.3 eV (∼530 nm) that emerges when copper atoms are incorporated into the 144-atom core.^[5]^


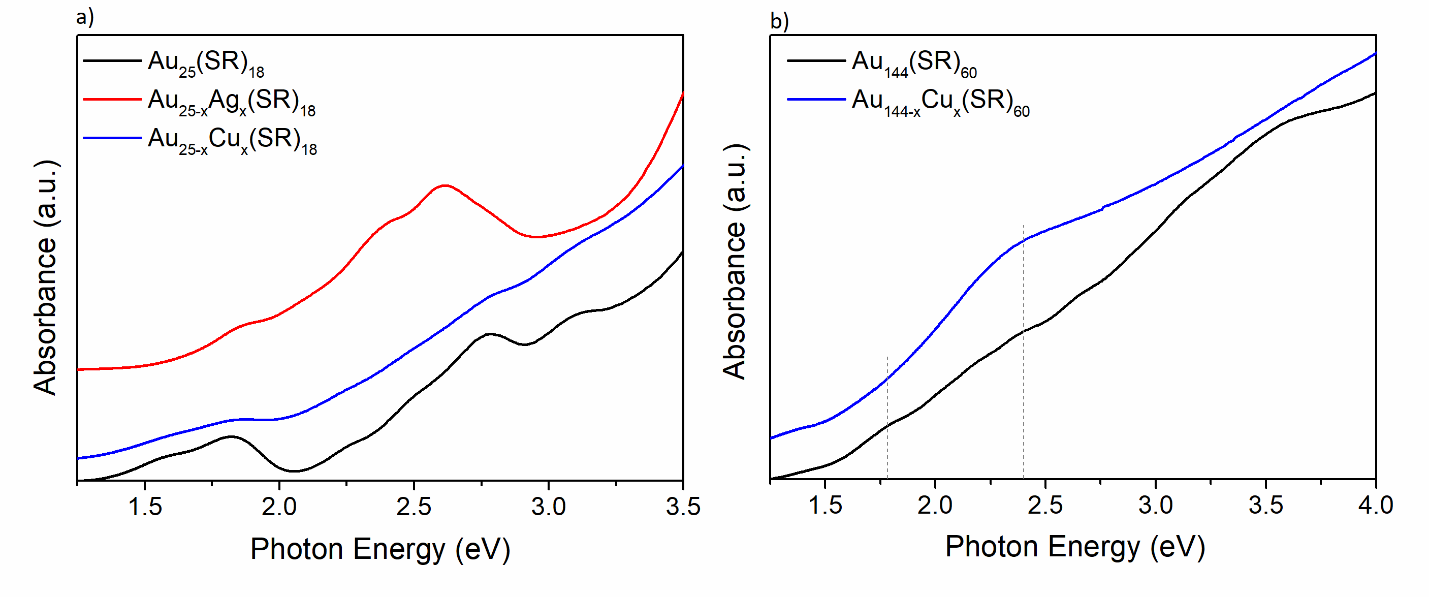


**Figure S1.** UV-vis absorption spectra of **a)** Au_25_(SR)_18_, Au_25–x_Ag_x_(SR)_18_ and Au_25–x_Cu_x_(SR)_18_; and b) Au_144_(SR)_60_ and Au_144–x_Cu_x_(SR)_60_. Note that for Au_25_(SR)_18_, the observed shoulder at ∼ 1.5 nm and the absorbance ratio A_3.11_/A_2.78_ = 1.17 have been attributed to the full reduction of the cluster (negative charge of -1 carrying a positive tetraoctylammonium, TOA^+^, counter ion).^[10,11]^

**Fig. S2** shows the MALDI mass spectra of pure Au_25_(SR)_18_ compared with bimetallic Au_25–_*_x_M_x_*(SR)_18_. The spectrum of Au_25_(SR)_18_ shows two main peaks at *m/z* 7393 and 6057, corresponding to the intact Au_25_(SR)_18_ cluster and the Au_21_(SR)_14_ fragment of Au_25_(SR)_18._^[12]^ The bimetallic *M*-doped Au NCs showed a distribution of peaks corresponding to diﬀerent numbers of Ag (up to 7) or Cu (up to 2) atoms doped into the 25-atom cluster as labeled directly above each peak at the top of **Fig. S2**. Note that all Au_25–_*_x_M_x_*(SR)_18_ NCs carry a negative charge with TOA^+^ as counterion, as confirmed by MALDI-MS (**Fig. S3**). The MALDI mass spectrum of Au_144_(SR)_60_ (**Fig. S4**) shows one broad peak centered at m/z ≈ 33 k, in agreement with the literature.^[1,5,9]^ No signal for Au_144–x_Cu_x_(SR)_60_ could be detected by MALDI-MS, probably due to the instability of the cluster under the high energy provided by the MALDI laser.

**Figure S2**. MALDI-MS of **left** Au_25_(SR)_18_ vs. Au_25–x_Ag_x_(SR)_18_, **right** Au_25_(SR)_18_ vs. Au_25–x_Cu_x_(SR)_18_, It can be seen that, by ESI-MS, only up to 2 Cu atoms seem to be doped in Au_25_(SR)_18_, this could be explained by a spontaneous de-alloying process occurring in solution over time,^[8]^ where initially formed Au_25–x_Cu_x_(SR)_18_ NCs can be converted to pure Au_25_(SR)_18_. This stability difference between Au_25–x_Cu_x_(SR)_18_ and Au_25–x_Ag_x_(SR)_18_ NCs has been observed in earlier works^[4,8,13]^ and has been attributed to the difference in the atomic radius of silver (1.44 Å, almost identical to that of Au) and Cu (1.28 Å).

**Figure S3.** Positive mode MALDI-MS of Au_25–_*_x_M_x_*(SR)_18_ showing the low m/s range of the counterion [TOA]^+^, where TOA^+^ = (C_8_H_17_)_4_N^+^.

**Figure S4.** MALDI-MS of Au_144_(SR)_60._ The mass deviation from the calculated value (*m/z* 36595.74) is due to a partial loss of ligands under the MALDI conditions.

**Cluster Catalysts Preparation**

To prepare the catalytic sample, the as-prepared NCs were loaded on carbon black (Vulcan XC-72R) with a mass ratio of 10% by mixing the NCs with carbon black in toluene and stirring overnight. Then, the solvent was removed by rotatory evaporation. The catalytic ink was prepared by dispersing the catalytic sample (5 mg) in isopropyl alcohol (500 μL) containing 40 μL of Nafion 117 solution (∼ 5%) from Sigma-Aldrich, and sonicated until a homogeneous ink was obtained. The working electrodes were prepared, drop-casting 40 μL of the as-prepared ink on each side of a gas diffusion layer of carbon paper (1.5 cm × 1 cm) (Spectracarb 2050–0550 from FuelCellStore) and allowed for solvent evaporation at room temperature. Only in the case of the study of the electrolyte cation effect (Figure S11), a glassy carbon electrode was used as working electrode by drop-casting 10 μL of the catalytic ink.

**Cluster Catalysts Characterization (XPS and TGA)**

The XPS spectra were measured on a SPECS spectrometer equipped with a Phoibos 150 MCD-9 detector using a nonmonochromatic X-ray source (Al) operating at 200 W. Samples were prepared by drop-deposition of a DCM cluster solution on a silicon chip. For the measurement of electrodes (Figure S9), carbon paper electrodes were prepared as described above but without Nafion to avoid the signal from sulfonate groups masking the signal from thiolate groups. The fitting of the experimental data to individual components was calculated from the area of the corresponding peaks after nonlinear background subtraction of the Shirley type.

**
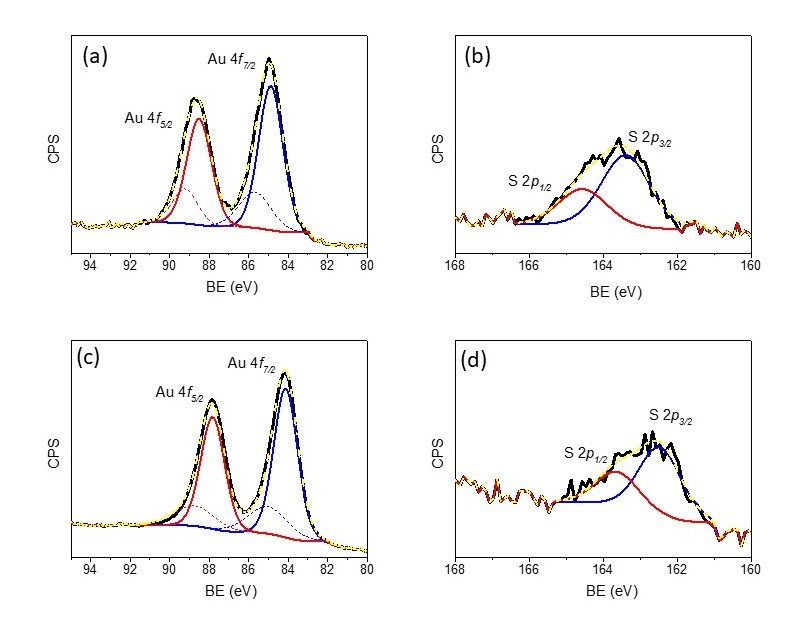
**

**Figure S5. (a)** Au 4f and (**b)** S 2p XPS spectra of Au_25_(SR)_18_. **(c)** Au 4f and (**d)** S 2p XPS spectra of Au_144_(SR)_60_. Experimental data (black line) are shown with envelopes (yellow-dotted line), and component fitting (red and blue lines). Red and blue solid lines in Au 4f spectra represent the components derived from core-Au(0) atoms, while red and blue dotted lines represent shell-Au(I) atoms.^[14,15]^ S 2p peaks at ∼163 eV are assigned to the gold-bound thiol groups.^[15,16]^


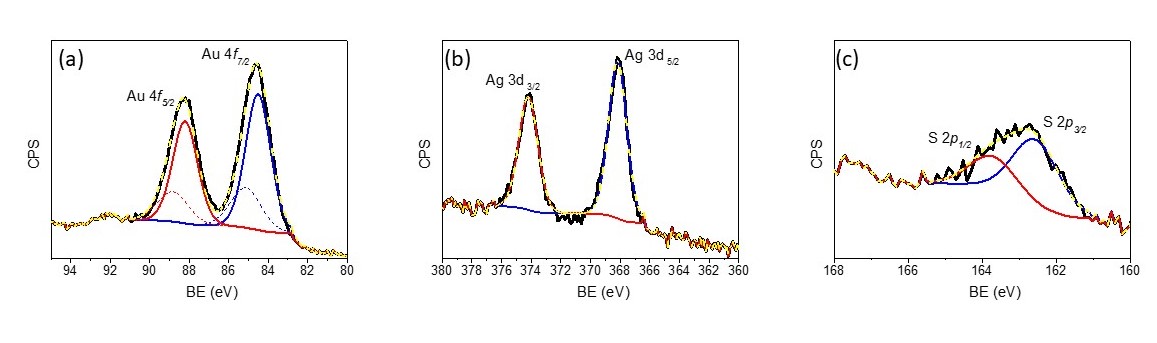


**Figure S6. (a)** Au 4f, **(b)** Ag 3d, and **(c)** S 2p XPS spectra of Au_25-x_Ag_x_(SR)_18_. Experimental data (black line) are shown with envelopes (yellow-dotted line), and component fitting (red and blue lines). Red and blue solid lines in Au 4f spectra represent the components derived from core-Au(0) atoms, while red and blue dotted lines represent shell-Au(I) atoms. S 2p peak at ∼163 eV is assigned to the gold-bound thiol groups. The Ag 3d peak (368.2 eV) indicates that the incorporated Ag is neutral.^[13]^


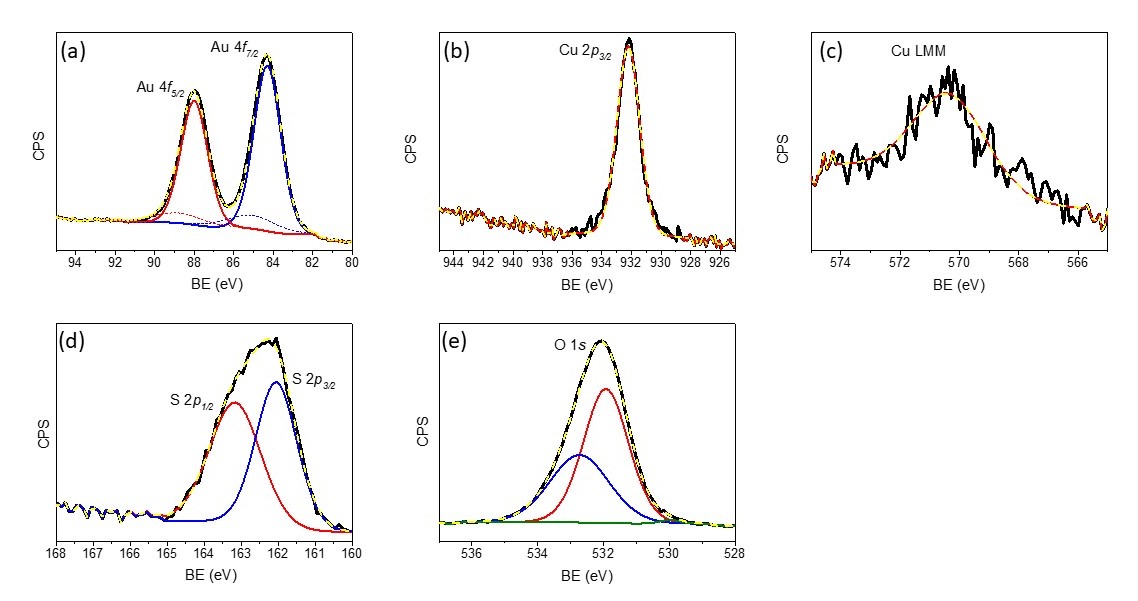


**Figure S7. (a)** Au 4f, **(b)** Cu 2p, **(c)** Cu LMM, **(d)** S 2p, and **(e)** O 1s XPS spectra of Au_25-x_Cu_x_(SR)_18_. Experimental data (black line) are shown with envelopes (yellow-dotted line), and component fitting (red, blue, and green lines). Red and blue solid lines in Au 4f spectra represent the components derived from core-Au(0) atoms, while red and blue dotted lines represent shell-Au(I) atoms. S 2p peak at ∼163 eV is assigned to the gold-bound thiol groups. Cu^0^ and Cu^+^ cannot be differentiated by the Cu 2p_3/2_ component (both located at ∼933 eV). However, the BE of the main Auger Cu LMM peak at 570.24 eV corresponds to Cu^+^.^[17]^ The O1s peak at 530 eV (green line) that corresponds to metal-O bonding^[17]^ is virtually absent, indicating that the amount of metal oxide is negligible.


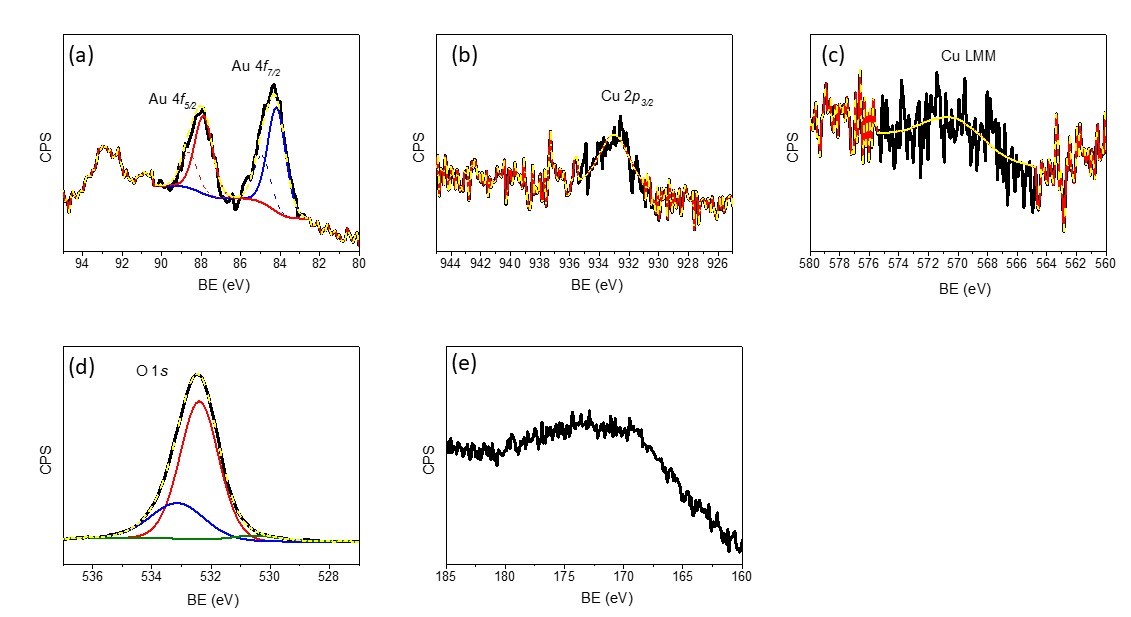


**Figure S8. (a)** Au 4f, **(b)** Cu 2p, **(c)** Cu LMM, **(d)** O 1s and **(e)** S 2p XPS spectra of of Au_144-x_Cu_x_(SR)_60_. Experimental data (black line) are shown with envelopes (yellow-dotted line), and component fitting (red, blue, and green lines). Red and blue solid lines in Au 4f spectra represent the components derived from Au(0), while red and blue dotted lines represent Au(I). Note the low intensity and poor-quality XPS spectra of Au_144-x_Cu_x_(SR)_60_; especially the S 2p region shows a broad band and the Au-S peak at ∼163 eV is not visible. Nevertheless, the presence of doped Cu is confirmed by the Cu 2p_3/2_ component. The BE of the main Auger Cu LMM peak at 570.30 eV indicates Cu^1+^. The amount of metal oxide is negligible since O1s peak at 530 eV (green line) is not significant.


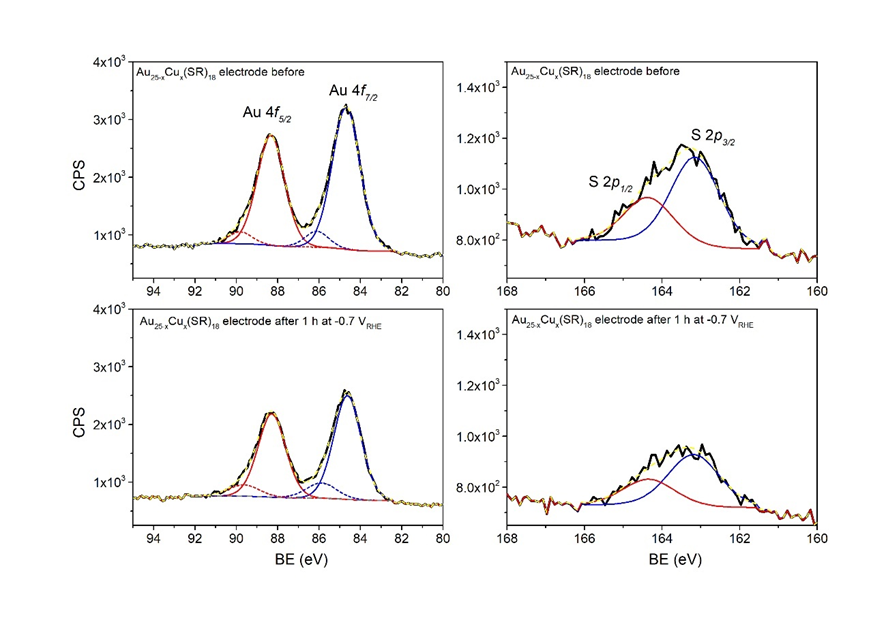


**Figure S9.** XPS profiles of carbon paper electrodes containing the Au_25–x_Cu_x_(SR)_18_ cluster before and after 1 h chronoamperimetric experiment at –0.7 V_RHE_. Note that the electrodes made for these measurements were prepared without the use of Nafion in order to avoid the signal from sulfonate groups masking the signal from thiolate groups.

**Table S1.** XPS compositional analysis of Au_25–_*_x_*Cu*_x_*(SR)_18_ electrodes before and after the chronoamperimetric experiment.

|  |  | Au 4f % At. Conc | S 2p % At. Conc | S : Au ratio |
| --- | --- | --- | --- | --- |
| BEFORE |  | 49.8 | 50.2 | 1.01 |
| AFTER |  | 55.5 | 44.5 | 0.80 |

Thermogravimetric analyses (TGA) were carried out in a Mettler Toledo TGA/SDTA 851e, with a temperature range from 20 to 1200 °C, a heating slope of 10 °C × min^-1^, and gas flux (air) of 20 mL × min^-1^.

**Figure S10.** TGA of NCs supported on carbon black. The indicated final weight % corresponds to {Au+*M*} content since under thermal conditions NCs lose surface ligands and transform to pure metal and the carbon black support burnt in the air atmosphere before 1000 °C.

**Electrocatalytic reaction studies**

The electrochemical characterization was carried out with an electrochemical workstation (VersaSTAT 3 Potentiostat Galvanostat). eCO_2_R was evaluated in a custom-made three-electrode H-cell configuration, separated by Nafion 117 membrane. The reference and counter electrodes used were Ag/AgCl KCl saturated and Pt coil, respectively. The potential (E_Ag/AgCl_ ) was converted to the reversible hydrogen electrode (RHE) scale (E_RHE_) using the following equation:^[18]^

$$E_{RHE}= E_{Ag/AgCl}+ 0.1976+0.059 \times pH$$

0.1 M KHCO_3_ (or MHCO_3_ (M = Na, K, Cs)) aqueous solution was used as the electrolyte. Catalytic performance tests were carried out using chronoamperometry (CA) measurements for 1 h at each potential (a fresh electrode was used for every potential). Current interrupt iR compensation was used in all experiments. Prior to the CA experiments, CO_2_ gas was bubbled into the cathode region of the H-cell for ∼20 minutes to ensure CO_2_ saturation (pH of CO_2_-saturated electrolyte = 6.81). Gas products were detected by gas chromatography (GC) using a Shimadzu GC-2014. H_2_ was quantified with a MolSieve 5 A column by TC detector, while CO was analyzed with a PorePlot Q column by FI detector. ^1^H nuclear magnetic resonance (NMR) spectroscopy (Bruker AV 300 MHz) was used for liquid product (HCOOH) detection. The faradaic efficiency of each reduction product of eCO_2_R was calculated as follows:

$$F_{i}\left( \% \right)=\frac{\left( n_{i}\times e_{i}\times F \right)}{Q}\times100$$

Where *n_i_* is the number of moles of the product produced, *e_i_* is the number of electrons required to generate the product, F is Faraday’s constant (96485 C / mol), and Q is the total charge consumed during CA.


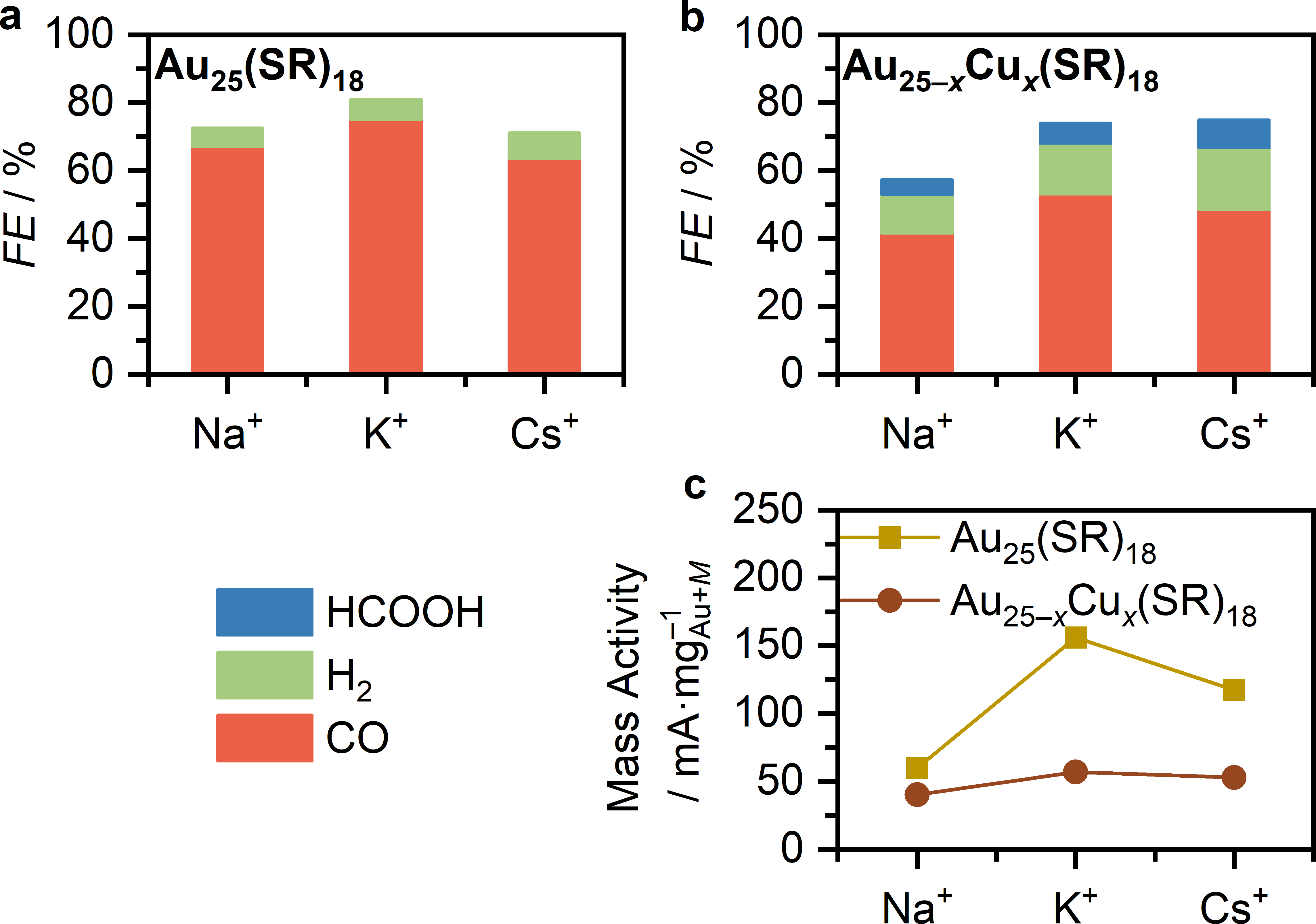


**Figure S11**. Electrolyte cation effect on the electrocatalytic CO_2_ reduction performance of Au_25–x_M_x_(SR)_18_ NCs on glassy carbon working electrode. Faradaic efficiencies of (a) Au_25_(SR)_18_; and (b) Au_25–x_Cu_x_(SR)_18_ at −0.7 V_RHE_ using electrolytes (MHCO_3_) containing different alkali cations. (c) Mass activities of the Au_25–x_M_x_(SR)_18_ NCs as a function of the alkali cations at −0.7 V_RHE_.


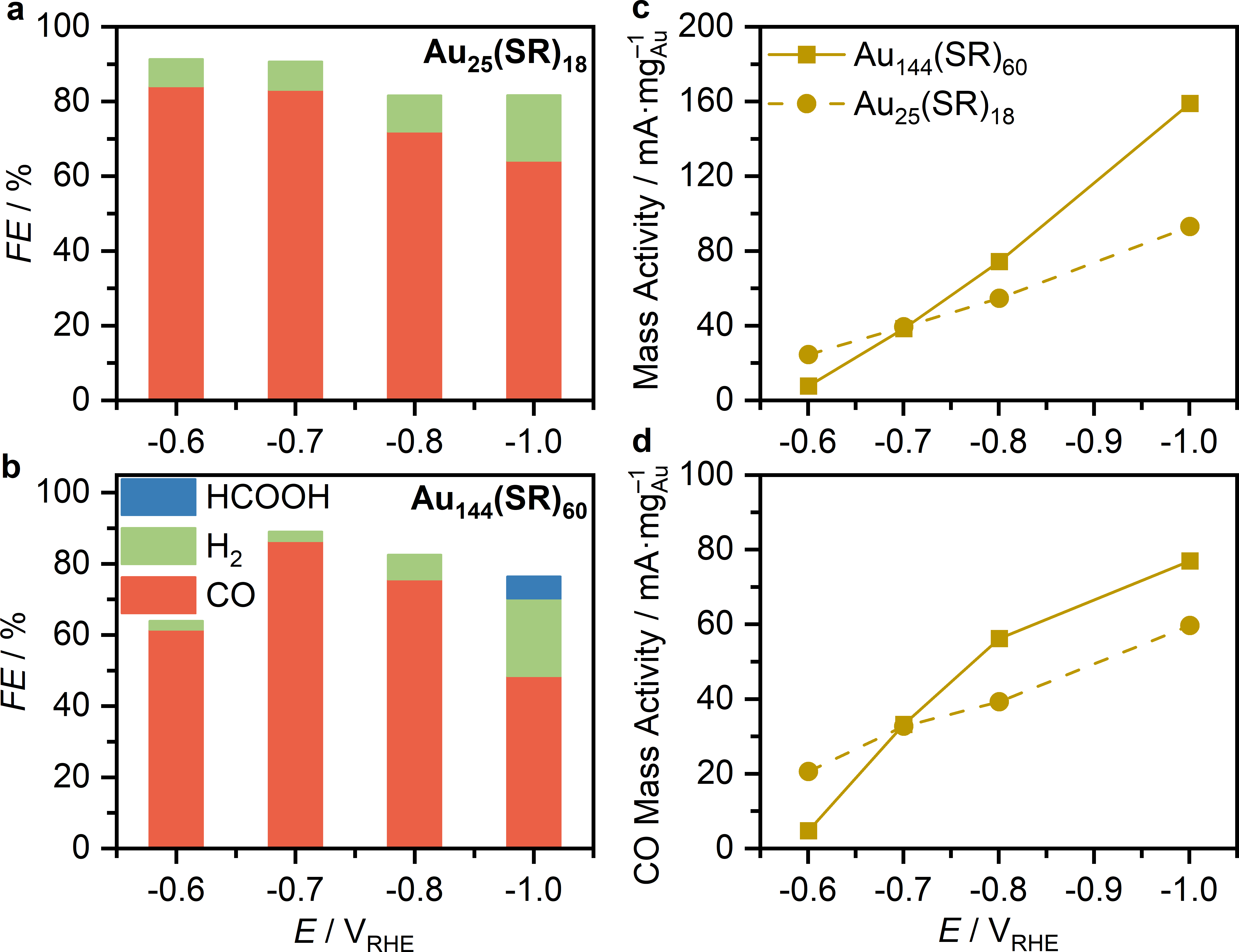


**Figure S12.** Electrochemical performance of Au_25_(SR)_18_ *vs.* Au_144_(SR)_60_ on carbon paper working electrodes: Faradaic efficiencies (a, b), mass activity (c), and partial CO mass activity (d) vs. applied potential.

**Figure S13.** ^1^H NMR spectra of the electrolyte extracted after the chronoamperometric experiments applying −1.0 V_RHE_. The chemical shift range for alkyl C-H protons is typically 0.5 – 2.5 ppm.^[19]^

**XAFS studies**

**Au L_3_-edge XAFS measurements** were carried out at NOTOS beamline at ALBA Synchrotron Light Source, Barcelona, Spain. The synchrotron light coming from a bending magnet has been first vertically collimated, then monochromatized using two pairs of liquid cooled Si(111) crystals and finally focused on the sample position down to ~500 × 500 μm^2^. A combination of Si and Rh stripe coating on the two optical mirrors guarantees the higher harmonics rejection. A 4 μm-thick Au foil (Goodfellow, 99.99%) has been used for calibrating the energy scale (Au L3-edge: 11919 eV), checking the alignment of scans, and determining the passive electron reduction factor in following data post-processing. Electrodes were prepared as described in section **Cluster Catalysts Preparation** above (supported nanoclusters onto carbon black with a mass ratio of 10% cast onto carbon paper working electrodes). XAS data on the electrodes were collected in fluorescence mode, which is highly sensitive to surface atoms, allowing us to specifically probe the nanoclusters (NCs) located on the surface. These surface NCs are directly involved in the eCO2RR. The XAS measurements were performed on these electrodes before and after the electrocatalytic reaction at various applied potentials. Data were collected using a 13 elements Si drift Canberra detector. The local structure of the samples has been then refined using the Extended X-ray absorption fine structure (EXAFS) signal in the k range 3:12 Å-1. Theoretical models for further data analysis have been obtained using the FEFF6 code and fitted to the EXAFS spectra by adjusting coordination number, distances, disorder factors. A two shell model (with Au-S and Au-Au contributions) has been considered to refine the data. To get more robust determination of these parameters, we performed a unique fit on the 15 spectra (including the Au foil). For each spectra of the dataset we considered as fitting parameters two coordination numbers CN (N_Au-S_, N_Au-Au_) and two correction factors for interatomic distances (δR_Au-S_ and δR_Au-Au_). Due to the limited k range available and the strong inter-correlation between the fitting parameters, we decided to use only two disorder parameters (𝜎^2^_Au-S_ and 𝜎^2^_Au-Au_) kept common for all the spectra in the dataset. This approach assures that the coordination numbers obtained from the analysis are consistent with all the experimental information available. Finally, we fit the energy correction to photoelectron reference ΔE0 and the passive electron reduction factors S02, again common to all the spectra.


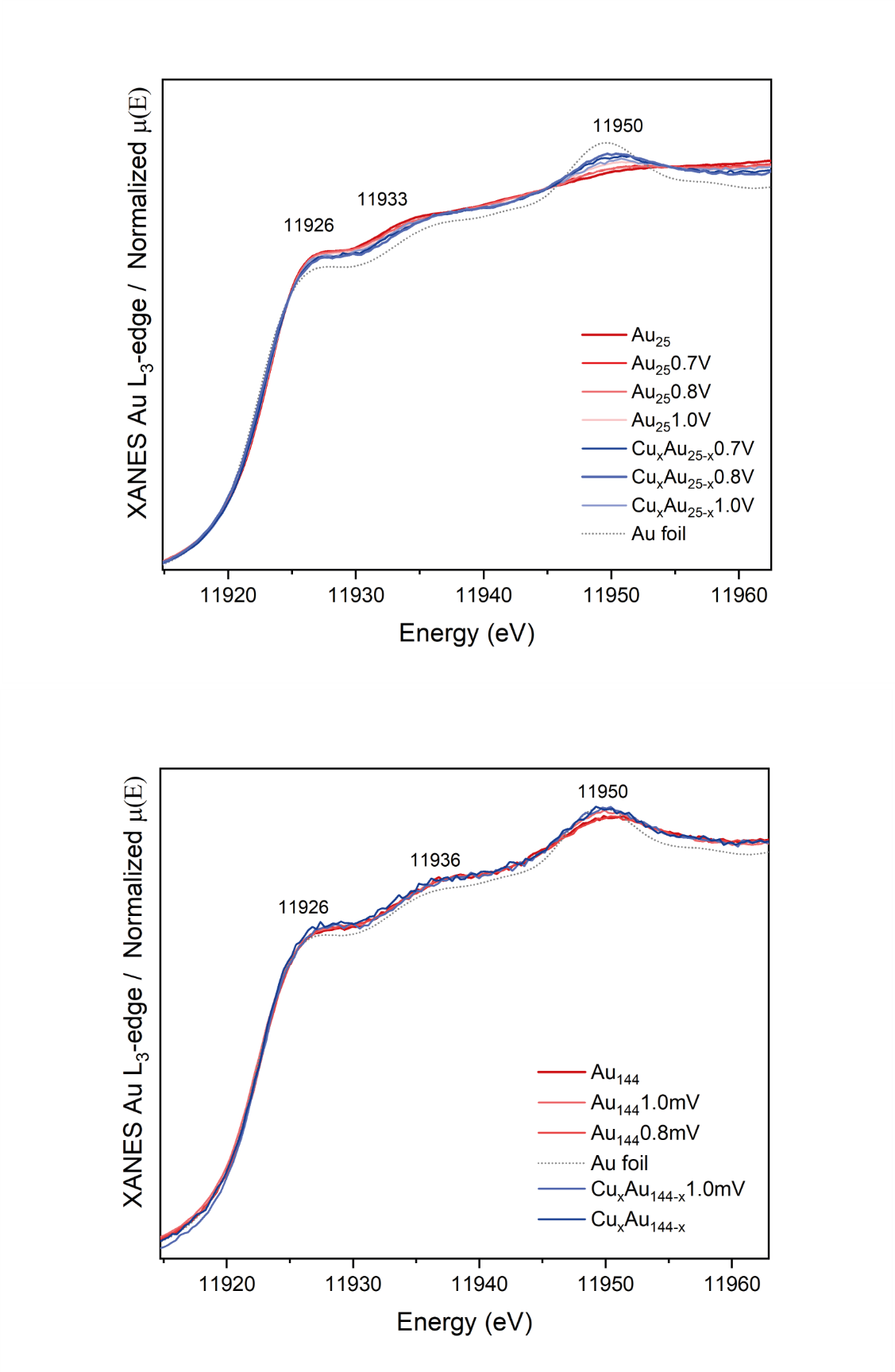


**Figure S14.** Au L_3_-edge X-ray absorption spectroscopy (XANES) of both monometallic and bimetallic clusters.


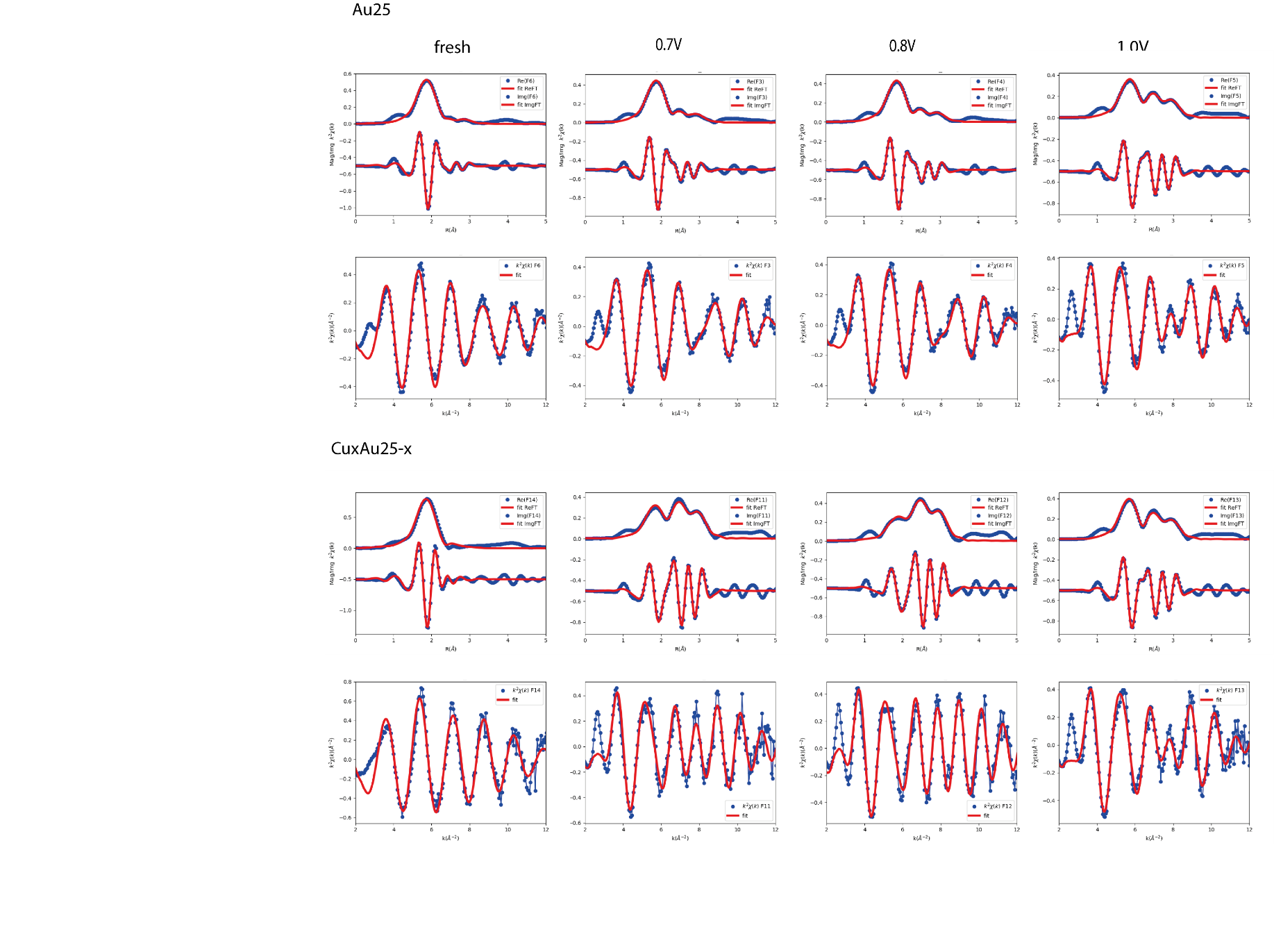


**Figure S15.** Representative examples of Au L_3_-edge fitting


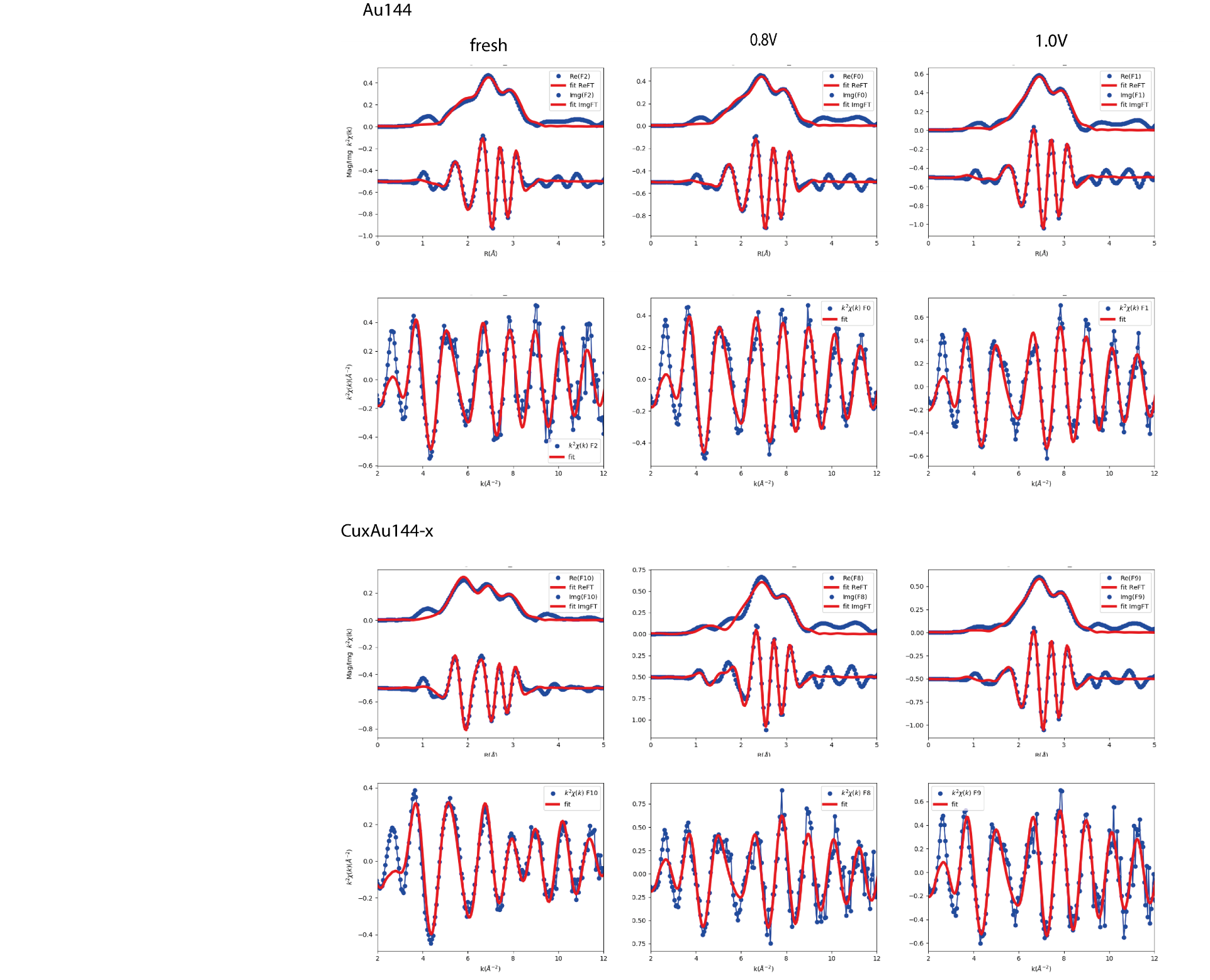


**Figure S16.** Representative examples of Au L_3_-edge fitting

**S K-edge XAFS measurements** were carried out on CLAESS beamline at ALBA Synchrotron Light Source, Barcelona, Spain.^[20]^ The synchrotron light coming from a multipole wiggler has been first vertically collimated, then monochromatized using two pairs of N_2_ liquid cooled Si(111) crystals and finally focused on the sample position down to ~500 × 500 μm^2^. Si stripes coatings and rejection angles of the two mirrors were opportunely chosen to guarantees the absence of high harmonics contributions into the spectra. Mo L_3_ edge has been used to calibrate energy scale. Measurements have been performed in fluorescence mode using a 6 channels Silicon Drift Detector SDD by Quantum Scientific.


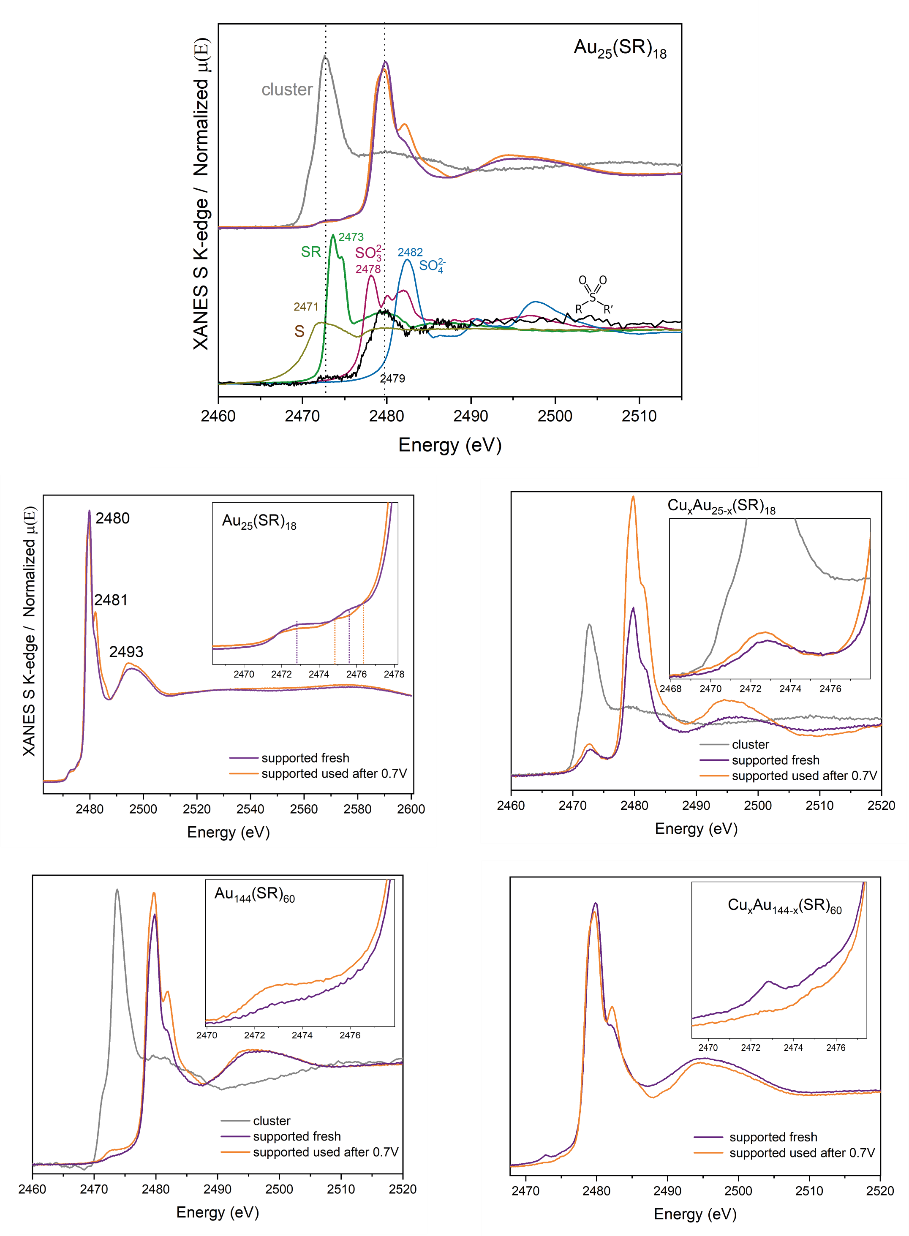


**Figure S17.** S K-edge XANES spectra of the unsupported clusters and the samples before and after electrocatalytic reaction (–0.7 V_RHE_) in addition to the references.

**COMPUTATIONAL DETAILS**

**DFT simulations**

Density Functional Theory (DFT) simulations were carried out using the Vienna Ab initio Simulation Package (VASP).^[21]^ The exchange functional used was the Perdew-Burke-Ernzerhof (PBE).^[22]^ Dispersion was included through the DFT-D2 method,^[21,23]^ with our reparametrized C_6_ coefficients.^[24]^ Inner electrons were represented through Projector Augment Wave (PAW) pseudopotentials,^[25,26]^ and the monoelectronic states for the valence electrons were expanded as plane waves with a kinetic energy cutoff of 450 eV.^[21]^ We sampled the Brillouin zone by a 1x1x1 Γ-centered k-points mesh from the Monkhorst-Pack method^[27]^ with a reciprocal grid size smaller than 0.03·2π Å^–1^ and a three-dimensional periodic cubic box with dimensions of 30 Å. For all the investigated systems, structures were relaxed using convergence criteria of 0.03 eV/Å and 10^−5^ eV for the ionic and electronic steps, respectively. Mechanistic studies were performed on the Au_25–_*_x_M_x_*(SR)_18_ and Au_144–_*_x_M_x_*(SR)_60_ (*M* = Au, Cu, Ag) bimetallic clusters, obtained by *M* substitution on atomically-precise Au_25_(SR)_18_ and Au_144_(SR)_60_ reported crystal structures.^[28–31]^ The organic part of (SR) ligands was replaced by -CH_3_ groups, as this approach has been shown before to be a computationally tractable method for modelling the reactivity and electronic structure of ligand-protected NCs.^[28,32]^ Potential energies of reaction intermediates were obtained using the gas-phase energies of CO_2_, H_2_O, and H_2_/H^+^ as thermodynamic sinks (Equations S3-6). The Computational Hydrogen Electrode (CHE) was used to obtain the relative energy between H^+^ and H_2_ at *U* = 0.0 V_RHE_ (Equation S7). ^[33,34]^

**Structural models**

The initial 3D Au_25_(SR)_18_ and Au_144_(SR)_60_ models were taken from crystal structures reported previously^[28–30,35]^ and optimized by means of DFT. Then, the bimetallic clusters models (Au_25–_*_x_M_x_*(SR)_18_ and Au_144–_*_x_M_x_*(SR)_60_) were generated by incorporating *M_x_* (*M* = Cu and Ag, *x* = 1-2) by substitution. For these metal dopant incorporations we considered staple positions, and icosahedral and rhombicosidodecahedral outer kernel positions of Au_25_*_–x_M_x_*(SR)_18_ and Au_144–_*_x_M_x_*(SR)_60_, respectively. Differential energies of first and second *M* incorporations (Δ*E_M_*) were obtained using Equations S1-2, where *E*_Au,bulk_ and *E_M_*_,bulk_ are the computed bulk energies normalized by the number of atoms per unit cell.

$\Delta E_{M}=E_{\text{Au}_{25-x}M_{x}\text{(SR)}_{18}}+E_{\text{Au,bulk}}-E_{\text{Au}_{25-(x-1)}M_{(x-1)}\text{(SR)}_{18}}-E_{M\text{,bulk}}$ (Eq. S1)

$\Delta E_{M}=E_{\text{Au}_{144-x}M_{x}\text{(SR)}_{60}}+E_{\text{Au,bulk}}-E_{\text{Au}_{144-(x-1)}M_{(x-1)}\text{(SR)}_{60}}-E_{M\text{,bulk}}$ (Eq. S2)

In the case of Au_25_*_–x_M_x_*(SR)_18_ all structures were computed as neutral (*q* = 0) and charged (*q* = –1) clusters, which is the more stable charge pair according to the redox potentials.^[36]^ The superatomic counting rule^[37]^ was used to assess the electron configuration of each cluster simulation.

**Energies of intermediates**

Potential energies of reaction intermediates were obtained using CO_2_, H_2_O, and H_2_/H^+^ as thermodynamic sinks (Equations S3-S5). The factor Δe*^−^* was applied to intermediates with an electron-enhanced adsorption, *e.g.* *CO_2_^−^ (Equation S6).

$x\text{CO}_{2}\text{ + * + (}z-2x)\text{H}_{2}\text{O + (}4x+y-2z)\text{H}^{+}\text{ + (}4x+y-2z)\text{e}^{-}+\Delta\text{e}^{-}\to\text{ *C}_{x}\text{H}_{y}\text{O}_{z}$ (Eq. S3)

$E_{\text{*C}_{x}\text{H}_{y}\text{O}_{z}}=E_{\text{DFT},\text{*C}_{x}\text{H}_{y}\text{O}_{z}}-E_{*}-(x)E_{\text{CO}_{2}}-(z-2x)E_{\text{H}_{2}\text{O}}-(4x+y-2z)E_{\text{H}^{+}}+n_{\text{e}^{-}}U$ (Eq. S4)

$n_{\text{e}^{-}}=\text{(}4x+y-2z)\text{ + }\Delta\text{e}^{-}$ (Eq. S5)

$*+\text{CO}_{2}+\text{e}^{-}\to\text{*CO}_{2}^{-}$ (Eq. S6)

The Computational Hydrogen Electrode (CHE)^[33,34]^ was used to obtain the relative energy of H^+^ from gas phase H_2_ computed energy at *U* = 0 V_RHE_, Equation S7.

$E_{\text{H}^{+}}=\frac{1}{2}E_{\text{H}_{2}}$ (Eq. S7)

**Ligand elimination energies**

Also, ligand elimination energies were obtained (considering *M*-S···R and further *M*···SH bond dissociations, or direct *M*···SR dissociations) using the computed gas phase energies of H_2_, CH_4_, HSCH_3_, and H_2_S molecules (Equations S8-13).

$M\text{-SR}+2(\text{H}^{+}+\text{e}^{-})\to M\text{-SH}+\text{CH}_{4}$ (Eq. S8)

$M\text{-SH}+(\text{H}^{+}+\text{e}^{-})\to M\text{-}+\text{H}_{2}\text{S}$ (Eq. S9)

$M\text{-SR}+(\text{H}^{+}+\text{e}^{-})\to M\text{-}+\text{HSCH}_{3}$ (Eq. S10)

$\Delta E_{M\text{-S···R}}=E_{M\text{-SH}}+E_{\text{CH}_{4}}-E_{M\text{-SR}}-2E_{\text{H}^{+}}+2U$ (Eq. S11)

$\Delta E_{M\text{···S(H)}}=E_{M\text{-}}+E_{\text{H}_{2}\text{S}}-E_{M\text{-SH}}-E_{\text{H}^{+}}+U$ (Eq. S12)

$\Delta E_{M\text{···S(R)}}=E_{M\text{-}}+E_{\text{HSCH}_{3}}-E_{M\text{-SR}}-E_{\text{H}^{+}}+U$ (Eq. S13)

**Table S2.** Energies (Δ*E_M_*) of *M* incorporation (*M* = Cu, Ag) to Au_25–_*_x_M_x_*(SR)_18_ cluster in kernel (k) and staple (s) positions. Second metal incorporation is evaluated both in the closest and farthest positions to the first metal as showed in Figure S22 (Position 1 and 2, respectively). For *x* = 2s,k and *x* = 2k,s the order means that first metal incorporates in staple and kernel, respectively. Energies of each metal bulk are used as references.

| *M* position | |  | *M* = Cu, Δ*E_M_* / eV | |  | *M* = Ag, Δ*E_M_* / eV | |
| --- | --- | --- | --- | --- | --- | --- | --- |
| 1rst | 2nd |  | Position 1 | Position 2 |  | Position 1 | Position 2 |
| k | - |  | –0.15 | |  | –0.18 | |
| k | k |  | –0.11 | –0.14 |  | –0.13 | –0.16 |
| k | s |  | –0.17 | –0.12 |  | –0.15 | –0.16 |
| s | - |  | 0.02 | |  | 0.14 | |
| s | s |  | –0.09 | 0.01 |  | 0.12 | 0.15 |
| s | k |  | 0.01 | 0.02 |  | 0.18 | 0.15 |

**Table S3.** Terminal and central ligand dethiolation energies (Δ*E_M_*_···S_) as H_2_S from Au_25–_*_x_M_x_*(SR)_16_(SH)_2_ cluster after S···R dissociation, and as HS-CH_3_ from Au_25–_*_x_M_x_*(SR)_18_ cluster. All results consider both neutral and charged clusters (*q* = 0, –1) and are at applied potential *U* = 0.0 V_RHE_. Computed energies of gas phase molecules H_2_S, HS-CH_3_, and H_2_/H^+^ are used as the references and Computational Hydrogen Electrode (CHE) approach^[33,34]^ is used to obtain the relative energy of H^+^ from gas phase H_2_ computed energy (see Ligand elimination energies in SI).

|  |  | Terminal ligand Δ*E_M_*_···S_ / eV | | | | |  | | Central ligand Δ*E_M_*_···S_ / eV | | | | |
| --- | --- | --- | --- | --- | --- | --- | --- | --- | --- | --- | --- | --- | --- |
|  |  | as H_2_S | |  | as HS-CH_3_ | | |  | as H_2_S | |  | as HS-CH_3_ | |
| *M_x_* |  | *q* = 0 | *q* = –1 |  | *q* = 0 | *q* = –1 | |  | *q* = 0 | *q* = –1 |  | *q* = 0 | *q* = –1 |
| Au |  | 0.83 | 1.34 |  | 1.02 | 1.49 | |  | 1.38 | 1.58 |  | 1.50 | 1.66 |
| Cu_1k_ |  | 1.23 | 1.75 |  | 1.45 | 1.91 | |  | 1.45 | 1.67 |  | 1.59 | 1.75 |
| Cu_1s_ |  | 0.82 | 1.47 |  | 1.06 | 1.62 | |  | 1.52 | 1.76 |  | 1.57 | 1.81 |
| Ag_1k_ |  | 0.98 | 1.51 |  | 1.12 | 1.61 | |  | 1.38 | 1.56 |  | 1.50 | 1.64 |
| Ag_1s_ |  | 0.72 | 1.31 |  | 0.85 | 1.41 | |  | 1.52 | 1.65 |  | 1.45 | 1.63 |

**Table S4.** First and second terminal ligands dethiolation energies (Δ*E_M_*_···SH_) on the same staple as H_2_S from Au_25–_*_x_M_x_*(SR)_16_(SH)_2_ cluster at applied potential of *U* = 0.0 V_RHE_. Computed energies of gas phase molecules H_2_S and H_2_/H^+^ are used as the references and Computational Hydrogen Electrode (CHE) approach^[33,34]^ is used to obtain the relative energy of H^+^ from gas phase H_2_ computed energy (see Ligand elimination energies in SI).

|  |  | Terminal ligand Δ*E_M_*_···SH_ / eV | |
| --- | --- | --- | --- |
| *M_x_* |  | 1_rst_ | 2_nd_ |
| Au |  | 0.83 | 1.70 |
| Cu_1k_ |  | 1.23 | 1.84 |
| Cu_1s_ |  | 0.82 | 1.79 |
| Ag_1k_ |  | 0.98 | 1.83 |
| Ag_1s_ |  | 0.72 | 1.69 |

**Table S5.** Bader charges (*q*_B_) of *M* on former staples (*M*-SR), S···R dissociated staples (*M*-SH), and staples with an eliminated terminal ligand (*M*···SH), and also for closest Au atom on kernel (Au_k_).

|  |  | *q*_B_ / \|e^–^\| | | | | |
| --- | --- | --- | --- | --- | --- | --- |
| *M* |  | *M*-SR | *M*-SH | *M*···SH |  | Au_k_ |
| Au |  | 0.08 | 0.08 | –0.02 |  | –0.08 |
| Cu |  | 0.42 | 0.42 | 0.35 |  | –0.15 |
| Ag |  | 0.31 | 0.32 | 0.25 |  | –0.12 |

**Table S6.** Adsorption energies (*E*_ads_) of *H on Au_25–_*_x_M_x_*(SR)_17_(SH) staple sites and on Au_25–_*_x_M_x_*(SR)_17_ kernel sites. All results consider both neutral and charged clusters (*q* = 0, –1) and are at applied potential *U* = 0.0 V_RHE_. Also, the number of superatomic counting rule electrons (*n*)^[37,38]^ is included to show that both on staples and kernel the lowest energies are obtained when *n* = 8. Computational Hydrogen Electrode (CHE) approach^[33,34]^ is used to obtain the relative energy of H^+^ from gas phase H_2_ computed energy (see Ligand elimination energies in SI).

|  |  | *H adsorption on staple site | | | | |  | *H adsorption on kernel site | | | | |
| --- | --- | --- | --- | --- | --- | --- | --- | --- | --- | --- | --- | --- |
|  |  | *q* = 0 | |  | *q* = –1 | |  | *q* = 0 | |  | *q* = –1 | |
| *M* |  | *E*_ads_ / eV | *n* |  | *E*_ads_ / eV | *n* |  | *E*_ads_ / eV | *n* |  | *E*_ads_ / eV | *n* |
| Au |  | 0.75 | 8 |  | 1.14 | 9 |  | –0.30 | 7 |  | –0.73 | 8 |
| Cu_1k_ |  | 0.72 | 8 |  | 1.05 | 9 |  | –0.57 | 7 |  | –1.01 | 8 |
| Cu_1s_ |  | 1.16 | 8 |  | 1.30 | 9 |  | –0.26 | 7 |  | –0.74 | 8 |
| Ag_1k_ |  | 0.77 | 8 |  | 1.16 | 9 |  | –0.41 | 7 |  | –0.87 | 8 |
| Ag_1s_ |  | 1.28 | 8 |  | 1.44 | 9 |  | –0.15 | 7 |  | –0.64 | 8 |

**Table S7.** Energies (Δ*E*) of first (RS-*M*···SR) and second (RS···*M*) ligand eliminations of Au_144–_*_x_M_x_*(SR)_60_ ligands as HS-CH_3_ and as H_2_S. All results are at applied potential *U* = 0.0 V_RHE_. Computed energies of gas phase molecules H_2_S, HS-CH_3_, and H_2_/H^+^ are used as the references and Computational Hydrogen Electrode (CHE) pproach^[33,34]^ is used to obtain the relative energy of H^+^ from gas phase H_2_ computed energy (see Ligand elimination energies in SI).

|  |  | Ligand elimination Δ*E* / eV | | | | |
| --- | --- | --- | --- | --- | --- | --- |
|  |  | as HS-CH_3_ | |  | as H_2_S | |
| *M*_s_ |  | RS-*M*_s_···SR | RS···*M*_s_ |  | HS-*M*_s_···SH | HS···*M*_s_ |
| Au |  | 0.81 | 1.00 |  | 0.87 | 1.08 |
| Cu_1s_ |  | 0.70 | 0.85 |  | 0.83 | 1.02 |
| Ag_1s_ |  | 0.48 | 0.51 |  | 0.66 | 0.65 |
| *M*_k_ |  | RS-Au···SR | RS··· Au |  | HS-Au ···SH | HS··· Au |
| Cu_1k_ |  | 1.12 | 0.67 |  | 1.19 | 0.76 |
| Ag_1k_ |  | 0.99 | 0.76 |  | 1.11 | 0.92 |

**Table S8.** Bite angle (*β*_n_) of RS-*M*-SR and HS-*M*-SH staples with *CO adsorbed on *M*, and distortion energy (*E*_distor_) of each of these staples to reach the bite angles shown for *M* = Cu (see Figure 6 and S21).

|  |  | *M* = Au | |  | *M* = Cu | |
| --- | --- | --- | --- | --- | --- | --- |
| cluster |  | *β*_n_ / ° | *E*_distor_ / eV |  | *β*_n_ / ° | *E*_distor_ / eV |
| Au_143_*M*(SR)_60_ |  | 182 | 0.77 |  | 142 | 0.53 |
| Au_143_*M*(SR)_58_(SH)_2_ |  | 183 | 1.04 |  | 121 | 0.65 |
| Au_24_*M*(SR)_18_ |  | 187 | 0.33 |  | 167 | 0.35 |
| Au_24_*M*(SR)_16_(SH)_2_ |  | 186 | 0.58 |  | 168 | 0.29 |

**
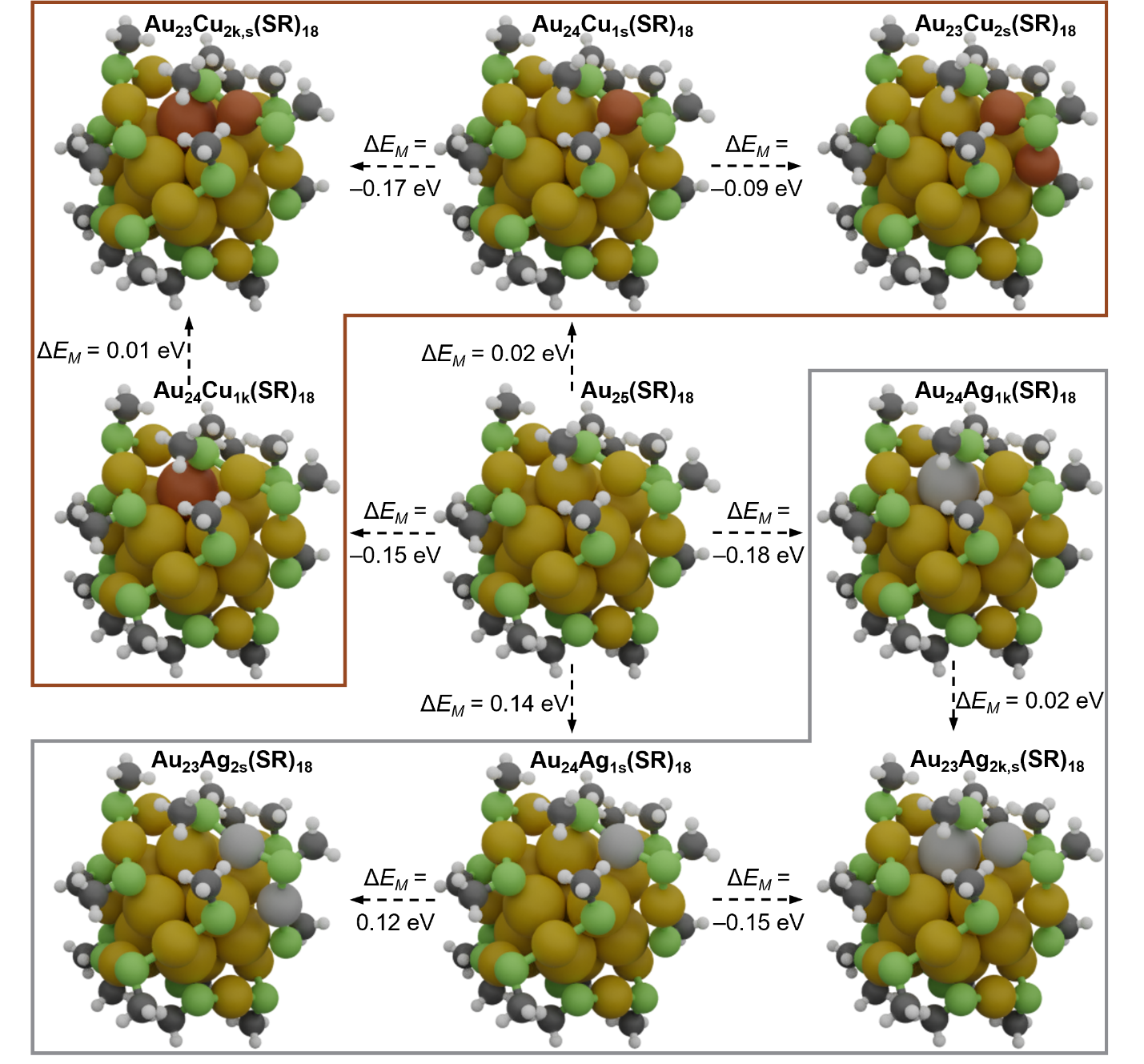
**

**Figure S18.** DFT-optimized Au_25_(SR)_18_ structure^[28,35]^ and Au_25–_*_x_M_x_*(SR)_18_ structures resulting from further *M_x_* incorporation (*M* = Cu, Ag and *x* = 1, 2) in kernel (k) and staple (s) positions. Differential energies of each metal dopant incorporation are included (Δ*E_M_*). Au, Cu, and Ag atoms are depicted in yellow, brown and grey, respectively, and smaller spheres are used to distinguish staple metal atoms than kernel ones. 2-phenyletanethiolate (PET) ligands are represented using methanethiolate ligands (SR), which is commonly used on DFT simulations^[28,35]^ and S, C, and H atoms are depicted in green, dark grey and white, respectively.


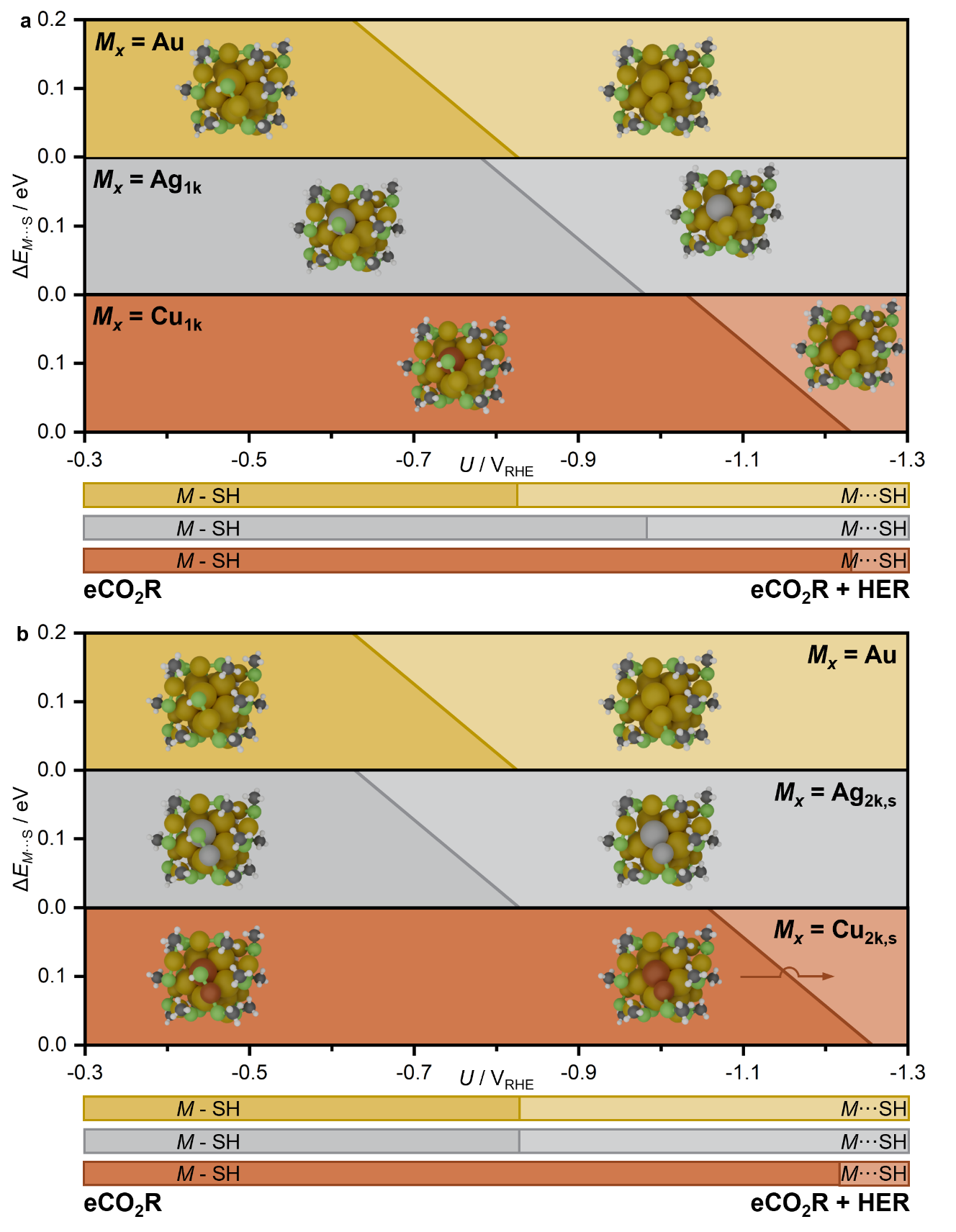


**Figure S19.** Phase diagrams of Au_25–_*_x_M_x_*(SR)_18_ ligand evolution under applied potential (*U*) for (**a**) *M_x_* = Au, Ag_1k_, and Cu_1k_; and for (**b**) *M_x_* = Au, Ag_2k,s_, and Cu_2k,s_. Dethiolation energies (Δ*E_M_*_···S_) are computed as a single *M*···SH bond dissociation on Au_25–_*_x_M_x_*(SR)_17_(SH) to form H_2_S and Au_25–_*_x_M_x_*(SR)_17_.


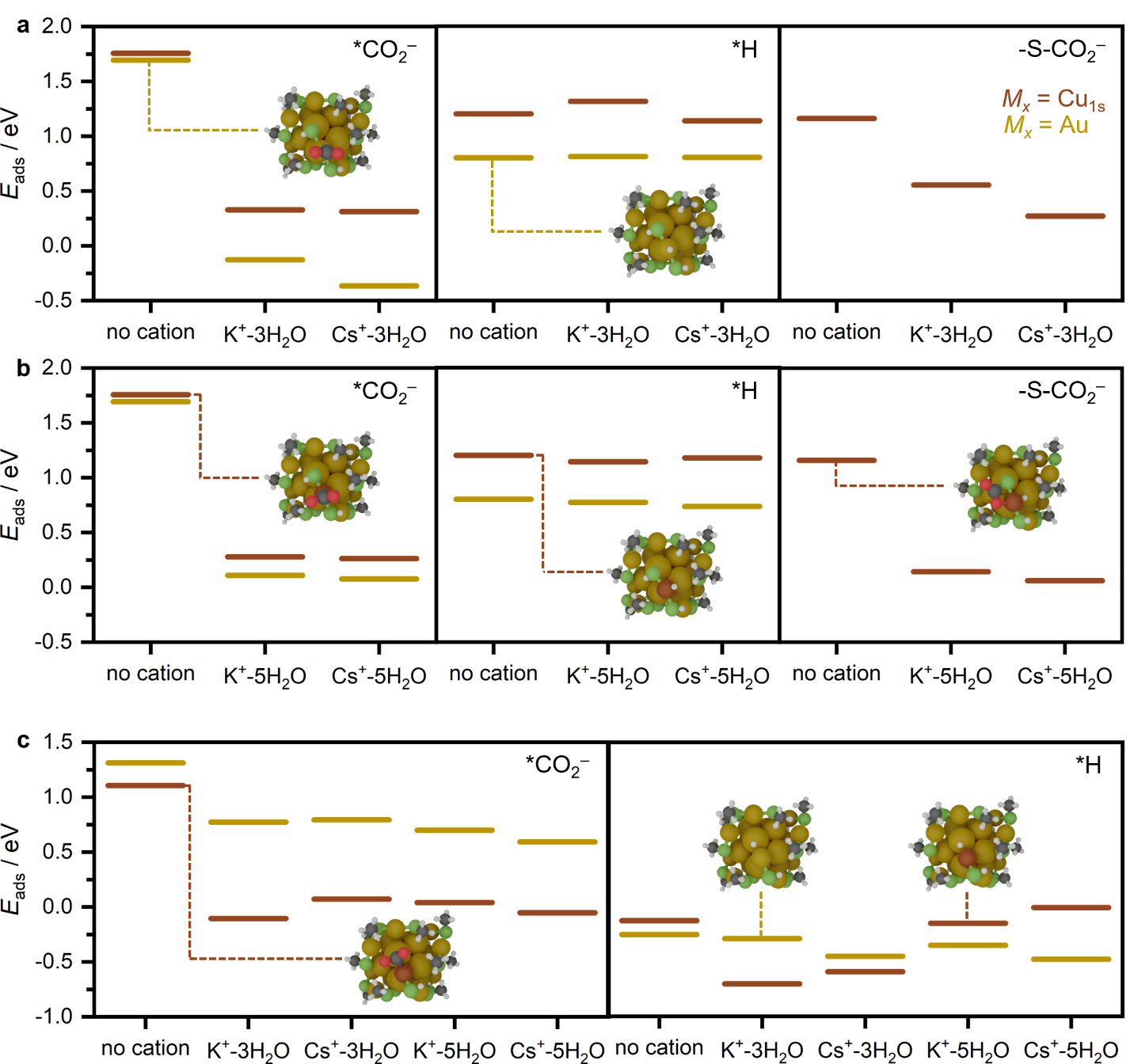


**Figure S20.** Adsorption energies (*E*_ads_) (**a**, **b**) of *CO_2_^–^, *H, and -S-CO_2_^–^ on Au_25–_*_x_M_x_*(SR)_16_(SH)_2_ staple sites (**c**) and of *CO_2_^–^ and *H on Au_25–_*_x_M_x_*(SR)_16_(SH) kernel sites with the effect of cations (K^+^ and Cs^+^) partially solvated with 3 H_2_O and 5 H_2_O molecules. All values are at applied potential of *U* = 0.0 V_RHE_. Au, Cu, S, C, O, and H atoms are depicted in yellow, brown, green, dark grey, red, and white, respectively.


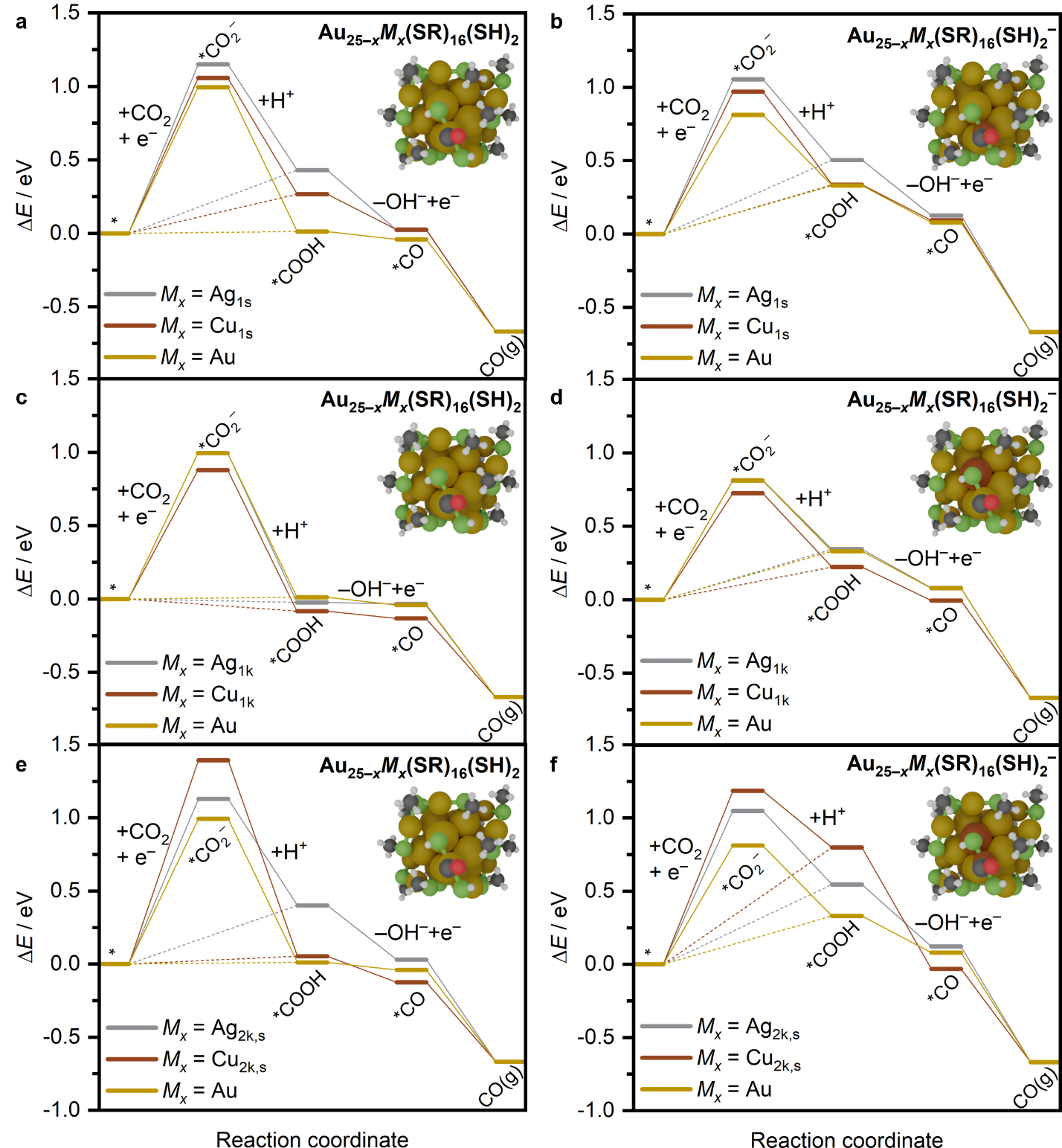


**Figure S21.** Energy profiles for eCO_2_R reaction to CO on staple sites of (**a**, **c**, **e**) Au_25–_*_x_M_x_*(SR)_16_(SH)_2_ cluster and (**b**, **d**, **f**) Au_25–_*_x_M_x_*(SR)_16_(SH)_2_^–^ charged cluster at *U* = –0.7 V_RHE_. The same reaction mechanism is evaluated for metal dopants (**a**, **b**) in staple positions (*M_x_* = Ag_1s_, Cu_1s_), (**c**, **d**) in kernel positions (*M_x_* = Ag_1k_, Cu_1k_), and (**e**, **f**) in both kernel and staple positions (*M_x_* = Ag_2k,s_, Cu_2k,s_). In all panels, energy profile for Au_25_(SR)_16_(SH)_2_ is included as a reference. Optimized structures of (**a**, **c**, **e**) *M* = Au and (**b**, **d**, **f**) *M* = Cu with *CO intermediate are included. Au, Cu, S, C, O, and H atoms are depicted in yellow, brown, green, dark grey, red, and white, respectively.


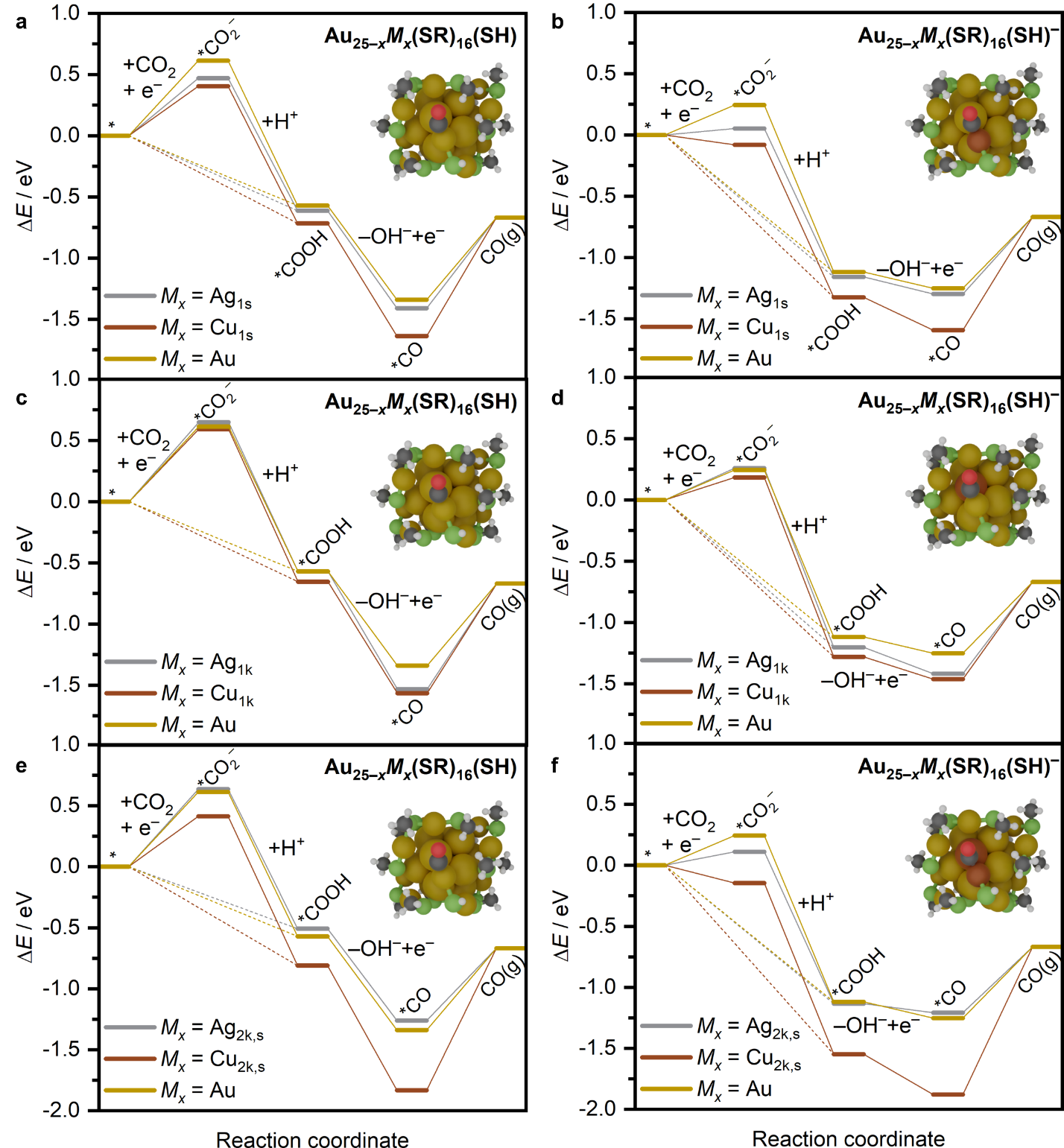


**Figure S22.** Energy profiles for eCO_2_R reaction to CO on kernel sites of (**a**, **c**, **e**) Au_25–_*_x_M_x_*(SR)_16_(SH) cluster and (**b**, **d**, **f**) Au_25–_*_x_M_x_*(SR)_16_(SH)^–^ charged cluster at *U* = –0.7 V_RHE_. The same reaction mechanism is evaluated for metal dopants (**a**, **b**) in staple positions (*M_x_* = Ag_1s_, Cu_1s_), (**c**, **d**) in kernel positions (*M_x_* = Ag_1k_, Cu_1k_), and (**e**, **f**) in both kernel and staple positions (*M_x_* = Ag_2k,s_, Cu_2k,s_). In all panels, energy profile for Au_25_(SR)_18_ is included as a reference. Optimized structures of (**a**, **c**, **e**) *M* = Au and (**b**, **d**, **f**) *M* = Cu with *CO intermediate are included. Au, Cu, S, C, O, and H atoms are depicted in yellow, brown, green, dark grey, red, and white, respectively.


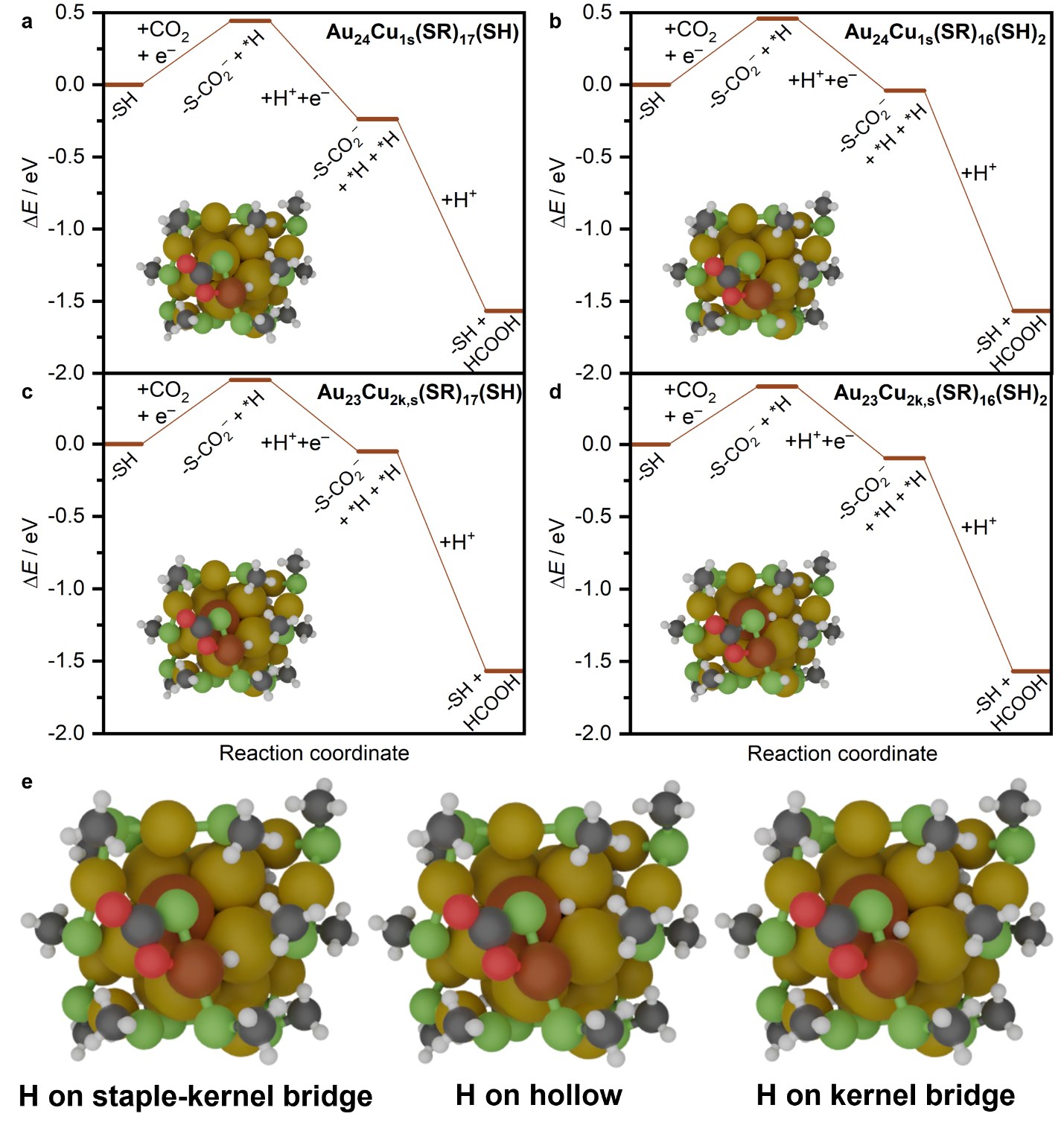


**Figure S23.** Energy profiles for eCO_2_R reaction to HCOO^–^ through thiocarbonate (-S-CO_2_^–^) intermediate on clusters with 1 Cu dopant (**a**) Au_24_Cu_1s_(SR)_17_(SH) and (**b**) Au_24_Cu_1s_(SR)_16_(SH)_2_, and with 2 Cu dopants (**c**) Au_23_Cu_2k,s_(SR)_17_(SH) and (**d**) Au_23_Cu_2k,s_(SR)_16_(SH)_2_ at *U* = –0.7 V_RHE_. (**e**) Optimized structures of -S-CO_2_^–^ intermediate on Au_23_Cu_2k,s_(SR)_17_(SH) cluster with 3 possible positions of H displaced from former -SH ligand. Inserts on each energy profile (**a**-**d**) represent the most stable configuration among the positions studied for displaced *H. Au, Cu, S, C, O, and H atoms are depicted in yellow, brown, green, dark grey, red, and white, respectively.

**
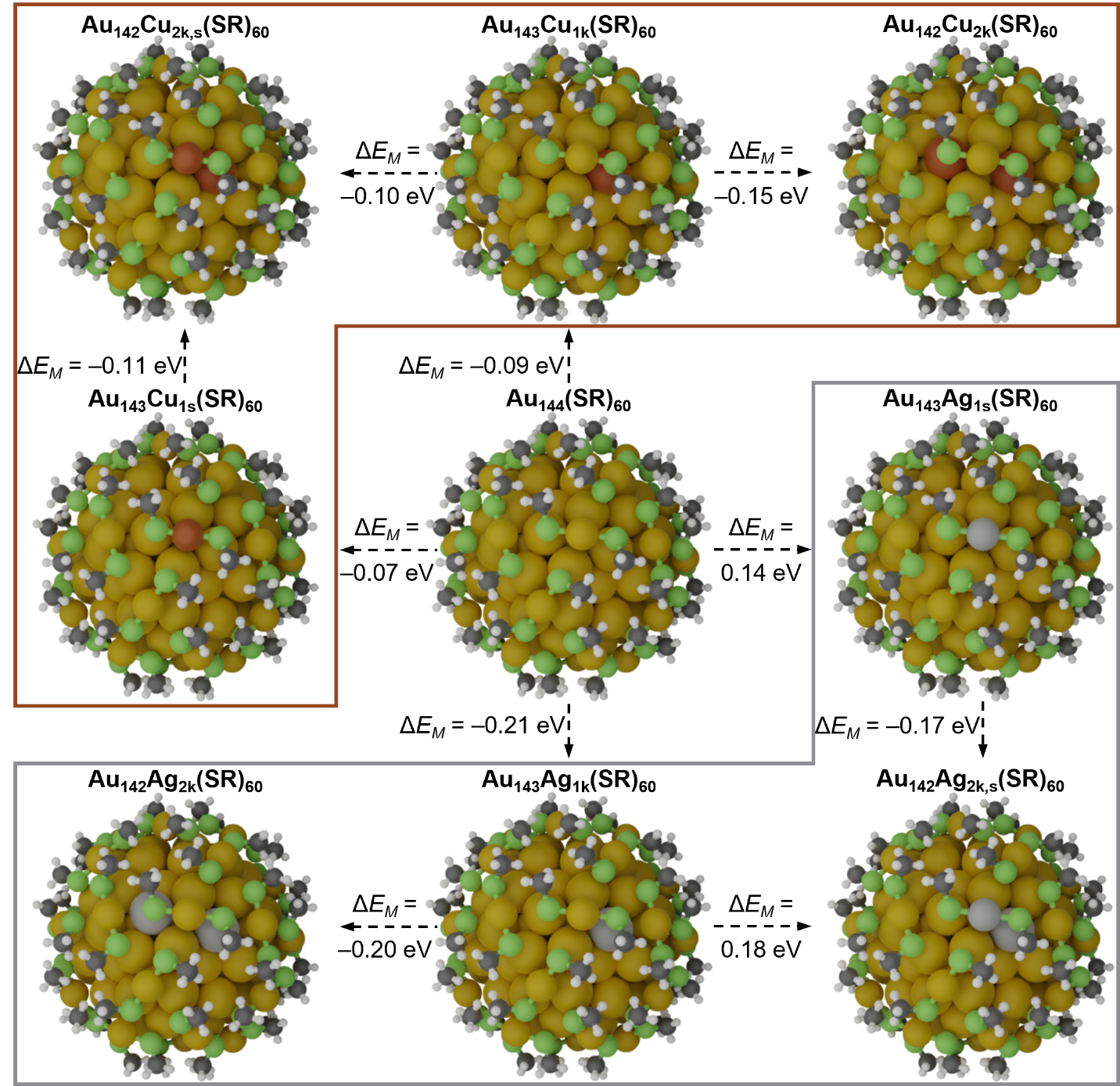
**

**Figure S24.** DFT-optimized Au_144_(SR)_60_ structure^[29,30]^ and Au_144–_*_x_M_x_*(SR)_60_ structures resulting from further *M_x_* incorporation (*M* = Cu, Ag and *x* = 1, 2) in kernel (k) and staple (s) positions. Differential energies of each metal dopant incorporation are included (Δ*E_M_*). Au, Cu, and Ag atoms are depicted in yellow, brown and grey, respectively, and smaller spheres are used to distinguish staple metal atoms than kernel ones. 1-dodecanethiol ligands are represented using methanethiolate ligands (SR), which is commonly used on DFT simulations^[28,35]^, and S, C, and H atoms are depicted in green, dark grey and white, respectively.


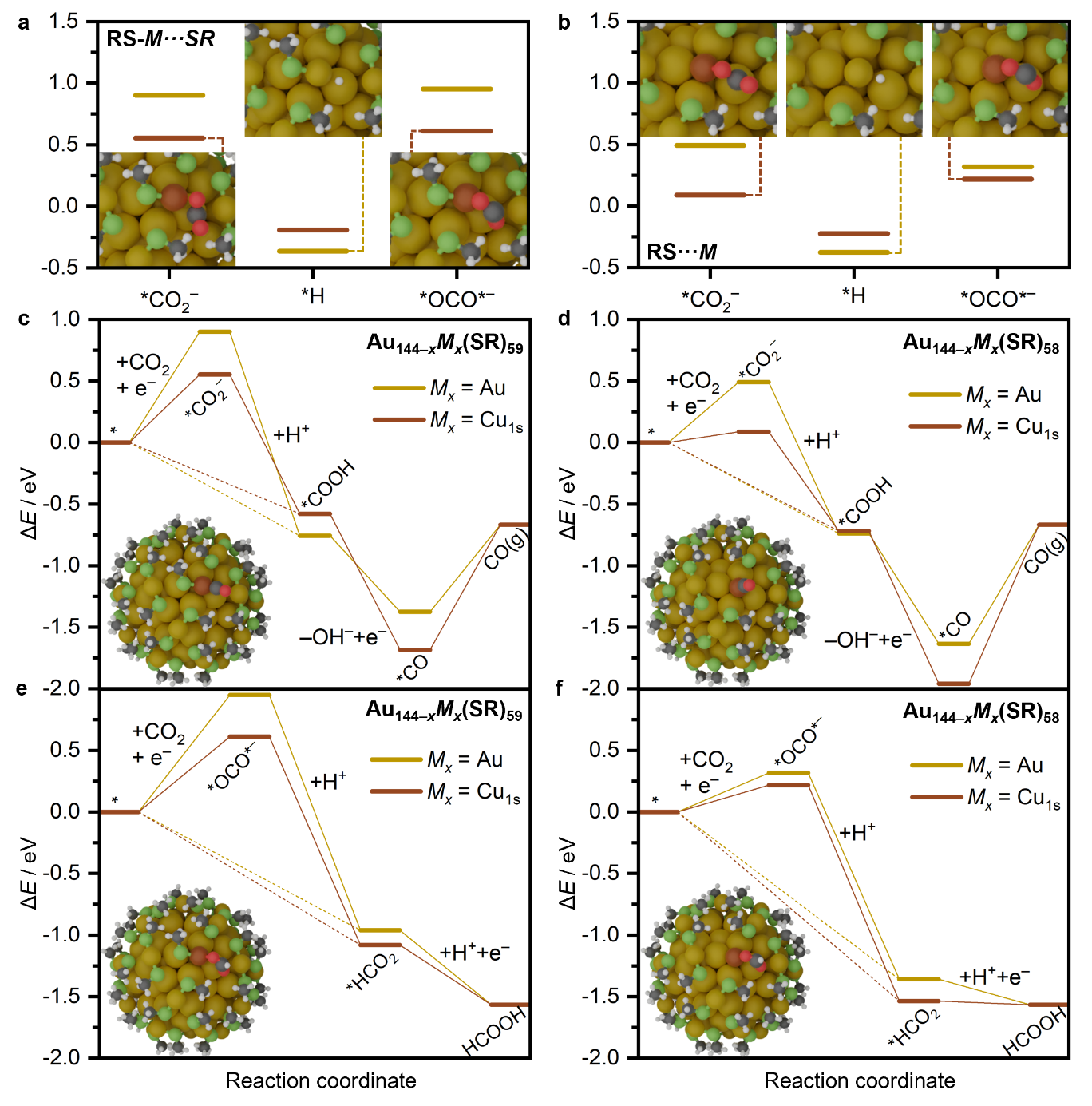


**Figure S25.** Adsorption energies of the first intermediates leading to CO, H_2_, and HCOOH products on (**a**) single-dethiolated (RS-*M*···SR) and (**b**) double dethiolated (RS···*M*) staples of Au_144–_*_x_M_x_*(SR)_60_ clusters (*M* = Au, Cu_1,s_ in yellow and brown respectively). Energy profiles for eCO_2_R reaction (**c**, **d**) to CO and (**e**, **f**) to HCOOH through *OCO*^–^ adsorption on kernel sites for (**c**, **e**) Au_144–_*_x_M_x_*(SR)_59_ and (**d**, **f**) Au_144–_*_x_M_x_*(SR)_59_ (*M_x_* = Au, Cu_1s_) at *U* = –0.7 V_RHE_. *CO_2_^–^ and *OCO*^–^ adsorption energies are computed at *U* = –0.7 V_RHE_, and *H adsorption energies are at *U* = 0.0 V_RHE_, and inserted panels depict the most stable structure for each adsorption. In each energy profile, the optimized structures of *M* = Cu cluster with *CO and *HCO_2_ intermediates are included. Au, Cu, S, C, O, and H atoms are depicted in yellow, brown, green, dark grey, red, and white, respectively.


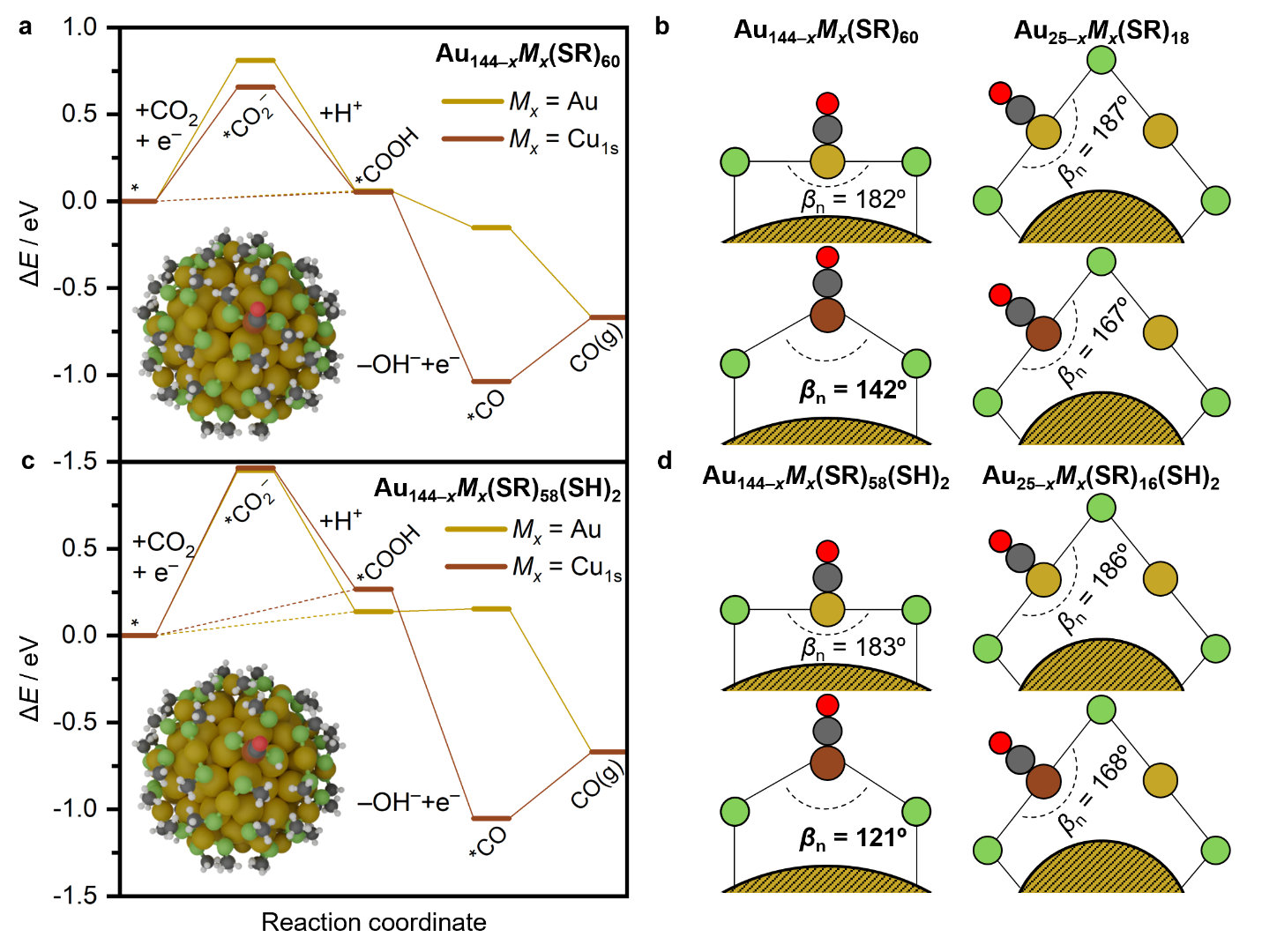


**Figure S26.** Energy profiles for eCO_2_R reaction to CO on staple sites (*M_x_* = Au, Cu_1s_) and schematic representation of staples bite angle (*β*_n_) bending for (**a**, **b**) Au_144–_*_x_M_x_*(SR)_60_ cluster and (**c**, **d**) Au_144–_*_x_M_x_*(SR)_58_(SH)_2_ cluster at *U* = –0.7 V_RHE_. In both energy profiles, the optimized structures of *M* = Cu cluster with *CO intermediate are included. In schematic representations of RS-*M*-SR staples, the *M*_2_(SR)_3_ staples from Au_25–_*_x_M_x_*(SR)_18_ are included at the right to show the higher capacity of Cu atoms in Au_144–_*_x_M_x_*(SR)_60_ staples to bend the bite angle. Yellow circle segments with stripped lines represent the kernel of each cluster and -H and -CH_3_ groups are not included in -S ligands for clarity. Au, Cu, S, C, O, and H atoms are depicted in yellow, brown, green, dark grey, red, and white, respectively.

**
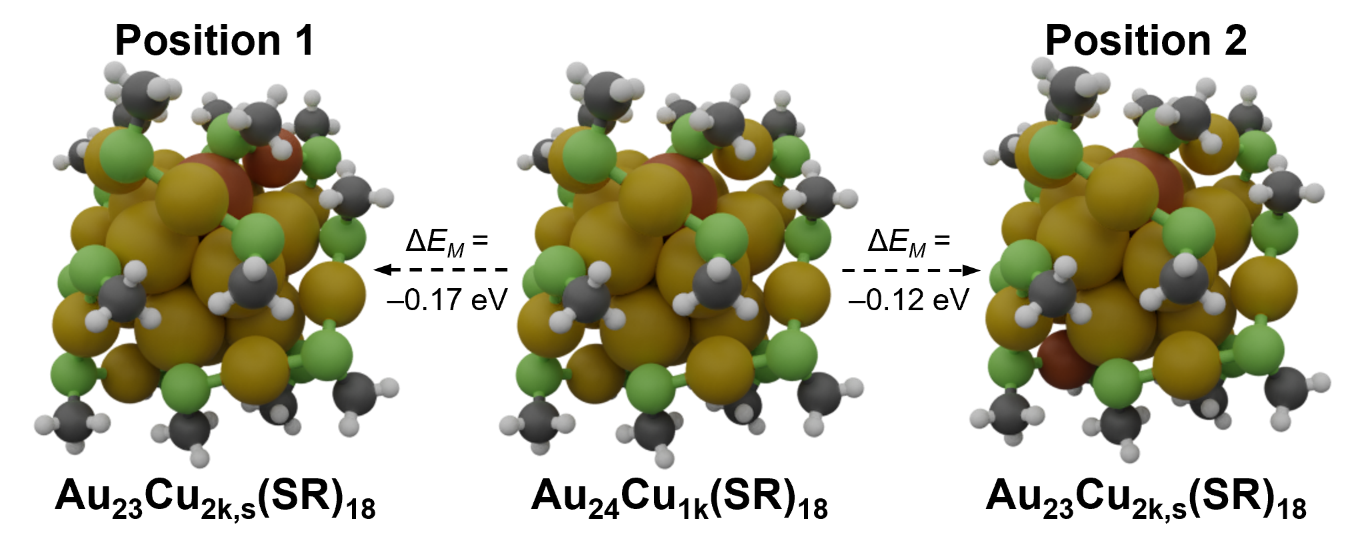
**

**Figure S27.** Incorporation of second *M* atom (*M* = Cu) in Au_24_Cu_1k_(SR)_18_ at the closest (Position 1) and farthest (Position 2) staple to form Au_23_Cu_2k,s_(SR)_18_, and incorporation energies.

**REFERENCES:**

[1] H. Qian, R. Jin, *Chem Mater* **2011**, *23*, 2209–2217.

[2] A. Shivhare, S. J. Ambrose, H. Zhang, R. W. Purves, R. W. J. Scott, *Chem Commun* **2012**, *49*, 276–278.

[3] E. Gottlieb, H. Qian, J. R. *Chem. Eur. J*. **2013**, *19*, 4238 – 4243.

[4] Y. Negishi, K. Munakata, W. Ohgake, K. Nobusada, *J Phys Chem Lett* **2012**, *3*, 2209–2214.

[5] A. C. Dharmaratne, A. Dass, *Chem Commun* **2014**, *50*, 1722–1724.

[6] Z. Wu, R. Jin, *Atomically Precise Metal Nanoclusters*, Springer International Publishing, **2021**.

[7] Y. Negishi, T. Iwai, M. Ide, *Chem Commun* **2010**, *46*, 4713-4715.

[8] E. Gottlieb, H. Qian, R. Jin, *Chem Eur J* **2013**, *19*, 4238–4243.

[9] H. Qian, R. Jin, *Nano Lett* **2009**, *9*, 4083–4087.

[10] D. R. Kauffman, D. Alfonso, C. Matranga, P. Ohodnicki, X. Deng, R. C. Siva, C. Zeng, R. Jin, *Chem Sci* **2014**, *5*, 3151–3157.

[11] J. F. Parker, J. E. F. Weaver, F. McCallum, C. A. Fields-Zinna, R. W. Murray, *Langmuir* **2010**, *26*, 13650–13654.

[12] A. Dass, A. Stevenson, G. R. Dubay, J. B. Tracy, R. W. Murray, *J Am Chem Soc* **2008**, *130*, 5940–5946.

[13] S. Wang, Y. Song, S. Jin, X. Liu, J. Zhang, Y. Pei, X. Meng, M. Chen, P. Li, M. Zhu, *J Am Chem Soc* **2015**, *137*, 4018–4021.

[14] T. Ohta, M. Shibuta, H. Tsunoyama, Y. Negishi, T. Eguchi, A. Nakajima, *J Phys Chem C* **2013**, *117*, 3674–3679.

[15] M. P. Casaletto, A. Longo, A. Martorana, A. Prestianni, A. M. Venezia, in *Surf Interface Anal*, **2006**.

[16] T. Ohta, M. Shibuta, H. Tsunoyama, Y. Negishi, T. Eguchi, A. Nakajima, *J Phys Chem C* **2013**, *117*, 3674–3679.

[17] L. Martin, H. Martinez, D. Poinot, B. Pecquenard, F. Le Cras, *J Phys Chem C* **2013**, *117*, 9, 4421–4430

[18] S. Niu, S. Li, Y. Du, X. Han, P. Xu, *ACS Energy Lett* **2020**, *5*, 1083–1087.

[19] *Handbook of Proton-Nmr Spectra and Data*, **1987**.

[20] L. Simonelli, C. Marini, W. Olszewski, M. Ávila Pérez, N. Ramanan, G. Guilera, V. Cuartero, K. Klementiev, *Cogent Physics* **2016**, *3*(1).

[21] G. Kresse, J. Furthmüller, *Phys Rev B Condens Matter Mater Phys* **1996**, B 54, 11169.

[22] J. P. Perdew, K. Burke, M. Ernzerhof, *Phys Rev Lett* **1996**, *77*, 3865.

[23] S. Grimme, *J Comput Chem* **2006**, *27*, 1787-1799.

[24] N. Almora-Barrios, G. Carchini, P. Błoński, N. López, *J Chem Theory Comput* **2014**, *10*, 11, 5002–5009.

[25] P. E. Blöchl, *Phys Rev B* **1994**, *50*, 17953.

[26] D. Joubert, *Phys Rev B Condens Matter Mater Phys* **1999**, *59*, 1758.

[27] K. Hu, M. Wu, S. Hinokuma, T. Ohto, M. Wakisaka, J. I. Fujita, Y. Ito, *J Mater Chem A Mater* **2019**, *7*, 2156-2164,

[28] J. Akola, M. Walter, R. L. Whetten, H. Häkkinen, H. Grönbeck, *J Am Chem Soc* **2008**, *130*, 12, 3756–3757.

[29] O. Lopez-Acevedo, J. Akola, R. L. Whetten, H. Grönbeck, H. Häkkinen, *J. Phys. Chem. C* **2009**, *113*, 13, 5035–5038.

[30] N. Yan, N. Xia, L. Liao, M. Zhu, F. Jin, R. Jin, Z. Wu, *Sci Adv* **2018**, 4 : eaat7259.

[31] N. Austin, S. Zhao, J. R. McKone, R. Jin, G. Mpourmpakis, *Catal Sci Technol* **2018**, *8*, 3795–3805.

[32] D. R. Alfonso, D. Kauffman, C. Matranga, *J Chem Phys* **2016**, *144*, 184705.

[33] J. K. Nørskov, J. Rossmeisl, A. Logadottir, L. Lindqvist, J. R. Kitchin, T. Bligaard, H. Jónsson, *Journal of Physical Chemistry B* **2004**, *108*, 46, 17886–17892.

[34] A. A. Peterson, F. Abild-Pedersen, F. Studt, J. Rossmeisl, J. K. Nørskov, *Energy Environ Sci* **2010**, *3*, 1311-1315.

[35] N. Austin, S. Zhao, J. R. McKone, R. Jin, G. Mpourmpakis, *Catal Sci Technol* **2018**, *8*, 3795–3805.

[36] O. López-Estrada, N. Mammen, L. Laverdure, M. M. Melander, H. Häkkinen, K. Honkala, *ACS Catal* **2023**, *13*, 13, 8997–9006.

[37] M. Walter, J. Akola, O. Lopez-Acevedo, P. D. Jadzinsky, G. Calero, C. J. Ackerson, R. L. Whetten, H. Grönbeck, H. Häkkinen, *Proc Natl Acad Sci U S A* **2008**, *105* (27) 9157-9162.

[38] G. Hu, Q. Tang, D. Lee, Z. Wu, D. E. Jiang, *Chem Mater* **2017**, 29, 11, 4840–4847.
